# Discovery of IHMT-MST1-39 as a novel MST1 kinase inhibitor and AMPK activator for the treatment of diabetes mellitus

**DOI:** 10.1038/s41392-023-01352-4

**Published:** 2023-04-05

**Authors:** Junjie Wang, Ziping Qi, Yun Wu, Aoli Wang, Qingwang Liu, Fengming Zou, Beilei Wang, Shuang Qi, Jiangyan Cao, Chen Hu, Chenliang Shi, Qianmao Liang, Li Wang, Jing Liu, Wenchao Wang, Qingsong Liu

**Affiliations:** 1 Anhui Province Key Laboratory of Medical Physics and Technology, Institute of Health and Medical Technology, Hefei Institutes of Physical Science, Chinese Academy of Sciences, Hefei, Anhui 230031 P. R. China; 2 University of Science and Technology of China, Hefei, Anhui 230026 P. R. China; 3 Hefei Cancer Hospital, Chinese Academy of Sciences, Hefei, Anhui 230031 P. R. China; 4 Precision Medicine Research Laboratory of Anhui Province, Hefei, Anhui 230088 P. R. China

**Keywords:** Drug discovery, Endocrine system and metabolic diseases

## Abstract

Insulin-producing pancreatic β cell death is the fundamental cause of type 1 diabetes (T1D) and a contributing factor to type 2 diabetes (T2D). Moreover, metabolic disorder is another hallmark of T2D. Mammalian sterile 20-like kinase 1 (MST1) contributes to the progression of diabetes mellitus through apoptosis induction and acceleration of pancreatic β cell dysfunction. AMP-activated protein kinase (AMPK) is an energy sensing kinase and its activation has been suggested as a treatment option for metabolic diseases. Thus, pharmacological inhibition of MST1 and activation of AMPK simultaneously represents a promising approach for diabetes therapy. Here, we discovered a novel selective MST1 kinase inhibitor IHMT-MST1-39, which exhibits anti-apoptosis efficacy and improves the survival of pancreatic β cells under diabetogenic conditions, as well as primary pancreatic islets in an ex vivo disease model. Mechanistically, IHMT-MST1-39 activated AMPK signaling pathway in hepatocytes in vitro, combination of IHMT-MST1-39 and metformin synergistically prevented hyperglycemia and significantly ameliorated glucose tolerance and insulin resistance in diabetic mice. Taken together, IHMT-MST1-39 is a promising anti-diabetic candidate as a single agent or in combination therapy for both T1D and T2D.

## Introduction

Diabetes mellitus has become a global health problem with rising economic burden and increasing prevalence every year.^1^ Various pathological mechanisms are thought to contribute to the development and progression of diabetes mellitus.^2^ Pancreatic islets are important endocrine organs that regulate internal metabolic balance and, pancreatic β cells are the only source of insulin in the body.^3^ Impaired function or damage of islet cells, especially insulin-producing β cells, has been associated with diabetes mellitus.^3^ Today, unhealthy diet and lifestyle lead to excessive nutrition intake and physical hypofunction, which alter glucose metabolism and energy homeostasis, resulting in increased need for functional β cells to deal with the metabolic imbalance.^4,5^ Constant and massive demand for insulin lead to overwork, degeneration, eventual β cell death and subsequent reduction of insulin production, rendering the body unable to maintain glycemic level; ultimately developing into T2D^6,7^ Different from insulin-independent T2D, T1D is an autoimmune disorder that results from T cell-mediated destruction of the insulin-secreting β cells in the pancreatic islets.^8,9^ Although the apoptotic and regenerative pathways share some common regulatory networks at the cellular level and have been well studied, efficient therapies targeting the cause of functional failure of pancreatic β cell is lacking.

In diabetes mellitus, as a key regulator of pancreatic β cell apoptosis and dysfunction, MST1 is thought to be activated by a series of upstream stress signals, which induce auto-phosphorylation at multiple sites in the activation loop of MST1 kinase domain.^10^ Among these sites, auto-phosphorylation of Thr183 within the kinase subdomain VIII is a principal event leading to the MST1 activation.^11,12^ Studies showed that MST1 kinase activity is highly elevated upon cleavage and release of the N-terminal catalytic domain.^13,14^ Genetic disruption of these cleavage sites not only impairs MST1 kinase activity, but also abrogates its nuclear localization and its ability to trigger apoptosis, which lowers glycemia and restores β cell survival in diabetic mouse models.^10,15,16^

Studies from the last few decades indicated that under a wide range of cellular disturbances including damaged mitochondrial function, starvation, and pathological conditions, like diabetes and diabetes-associated complications, AMPK is responsible for coordinating the global cellular response to energetic stress so as to produce beneficial physiological changes needed.^17^ The hepatic de novo lipogenesis (DNL) and steatosis are inhibited when the AMPK pathway is activated in mice.^18^ AMPK activation has also been suggested as an approach to attenuate hepatic injury and fibrogenesis by inhibiting multiple pro-inflammatory signaling pathways.^19,20^ Taken together, enhancing AMPK activity provides an attractive and widely studied therapeutic option for a variety of diseases. In addition, it is worth noting that T2D is characterized by multiple manifestations, such as deterioration of renal function, visual impairment, and various other chronic complications, which are secondary complications arising from metabolic status imbalance.^21^ Therefore, regulating AMPK may improve metabolism status, including reduction in the production of triglyceride, cholesterol, and free fatty acid, which is generally considered as secondary markers in the management of diabetes mellitus.^22^

As delineated above, the feasibility of MST1 and AMPK as therapeutic targets has been demonstrated in pancreatic β cells and diabetic mouse models, and thus the development of MST1 inhibitors and AMPK activators could therefore provide a promising approach as pancreas protective and metabolism adjusting agents for the management of diabetes mellitus. In this study, we report a novel anti-diabetic agent IHMT-MST1-39, which not only blocks MST1 mediated apoptotic signaling cascade in pancreatic β cells, but also enhances AMPK activation with improved metabolic status. In addition, we confirmed the significant glycemic control effects of combining of IHMT-MST1-39 and metformin, as well as improving glucose tolerance and insulin resistance in rodent diabetic models. Therefore, we propose a new therapeutic strategy to protect pancreatic β cells and improve metabolic status that leverages a heretofore new glycemic regulation approach through simultaneous modulation of activities of MST1 and AMPK using a drug combination of IHMT-MST1-39 and metformin.

## Results

### Discovery of IHMT-MST1-39 as a potent and selective MST1 inhibitor

**Fig. 1:**
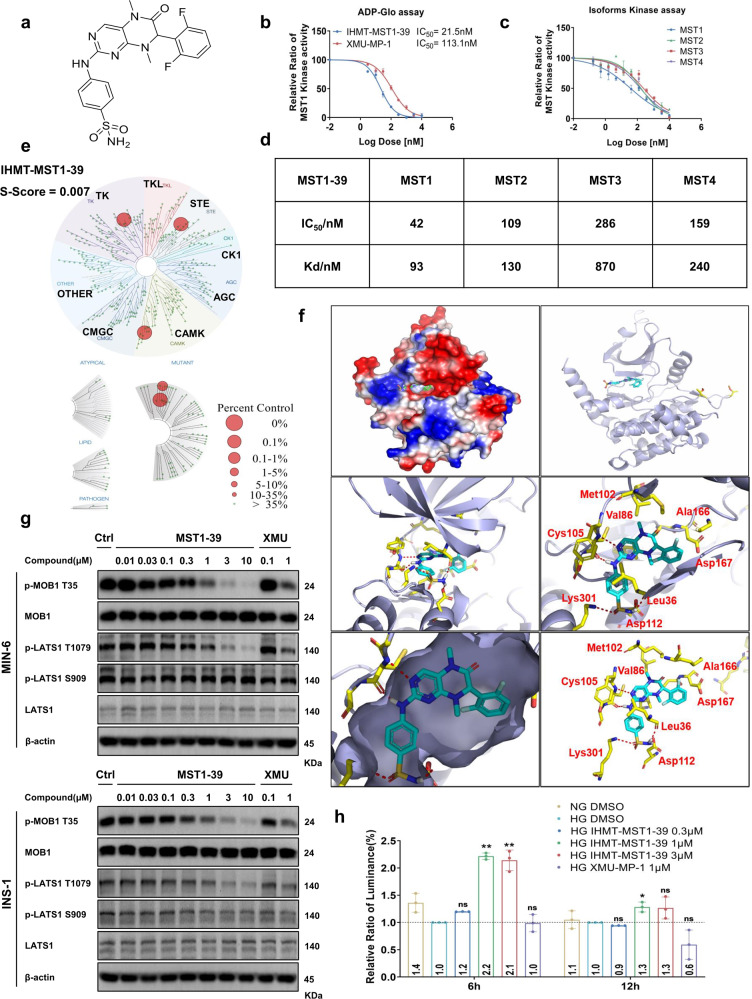
Discovery of IHMT-MST1-39 as a potent and selective MST1 inhibitor. **a** The chemical structure of IHMT-MST1-39. **b** ADP biochemical assay determination of IC_50_ of IHMT-MST1-39 and XMU-MP-1 against MST1 at different compound concentrations. **c**, **d** IC_50_ and KD values of IHMT-MST1-39 against different MST isoforms. Data were expressed ± SEM from three independent experiments (*n* = 3). **e** The KINOME-scan result of IHMT-MST1-39 against a panel of 468 kinases. **f** Molecular modeling analyses of the binding mode of MST1 with IHMT-MST1-39. MST1 homology model, template PDB code:6yat. **g** Inhibitory effects against the phosphorylation of MOB1, LATS1 in rodent pancreas cell lines after treatment of compound for 2 h. **h** YAP-TEAD reporter activity was measured in MDA-MB-231 cells exposed to high glucose, and then treated with compound at different time, compound activities were compared to high glucose control

To develop a highly potent MST1 kinase inhibitor, we applied a drug design approach based on structure-activity relationship and discovered a potent MST1 inhibitor, IHMT-MST1-39 (Fig. 1a). IHMT-MST1-39 exhibited inhibitory activities, with IC_50_ values of 42 nM and 109 nM against MST1 and MST2 in biochemical assay, respectively (Fig. 1b–d). Kinase selectivity of IHMT-MST1-39 was further determined using DiscoveRx’s KinomScan^TM^ technology, profiling the inhibitor at a concentration of 1 μM against a panel of 468 diverse kinases using an ATP-competition binding assay in vitro. IHMT-MST1-39 exhibited good overall kinase selectivity with a selectivity score of 0.007 (Fig. 1e). Besides MST1 and MST2, it also showed binding affinity against a few other kinases, such as RSK4 and SNARK. In addition, computer-aided homology molecular modeling of the MST1/IHMT-MST1-39 structure (PDB ID: 6yat) revealed the possible formation of four hydrogen bonds between IHMT-MST1-39 and MST1 (Fig. 1f), which indicates that IHMT-MST1-39 binds directly to MST1 protein. Together, these data suggested that IHMT-MST1-39 is a potent and selective MST1 kinase inhibitor.

To verify the functional relevance of these interactions, we performed site-directed mutagenesis studies at these three residues. We found the inhibition potencies of IHMT-MST1-39 were remarkably reduced by MST1 mutations of D112A and K301A compared to wild-type kinase, which is consistent with our hypothesis above (Supplementary Fig. S1a–c). However, due to the critical role of Cys105 residue for MST1 kinase activity, mutation of C105Y dramatically compromised the kinase activity of MST1 and thus we did not observe effects of our compound on MST1 function (Supplementary Fig. S1a–c). Together, our structural analysis and mutagenesis studies confirmed our proposed binding mode of our molecule with MST1 protein.

Because IHMT-MST1-39 targets MST1/2 in the Hippo signaling pathway, we next assessed the phosphorylation of MOB1 and LATS1 (Fig. 1g), which are known downstream substrates of MST1/2, and we found that IHMT-MST1-39 treatment in rodent pancreas cell lines, MIN-6 and INS-1, indeed lead to decrease in the phosphorylation levels of both MOB1 and LATS1.

To validate the effect, we further explored whether inhibition of MST1/2 in MDA-MB-231 cells would activate YAP, a main effector of the Hippo Pathway. Using TEAD luciferase reporter system,^23^ we detected a significant increase in YAP activity after IHMT-MST1-39 treatment in high glucose condition (Fig. 1h), indicating that the compound treatment could inhibit MST1/2 activity in MDA-MB-231 cells and subsequently upregulate YAP activity.

### IHMT-MST1-39 blocks pancreatic β cell apoptosis through MST1 inhibition

**Fig. 2:**
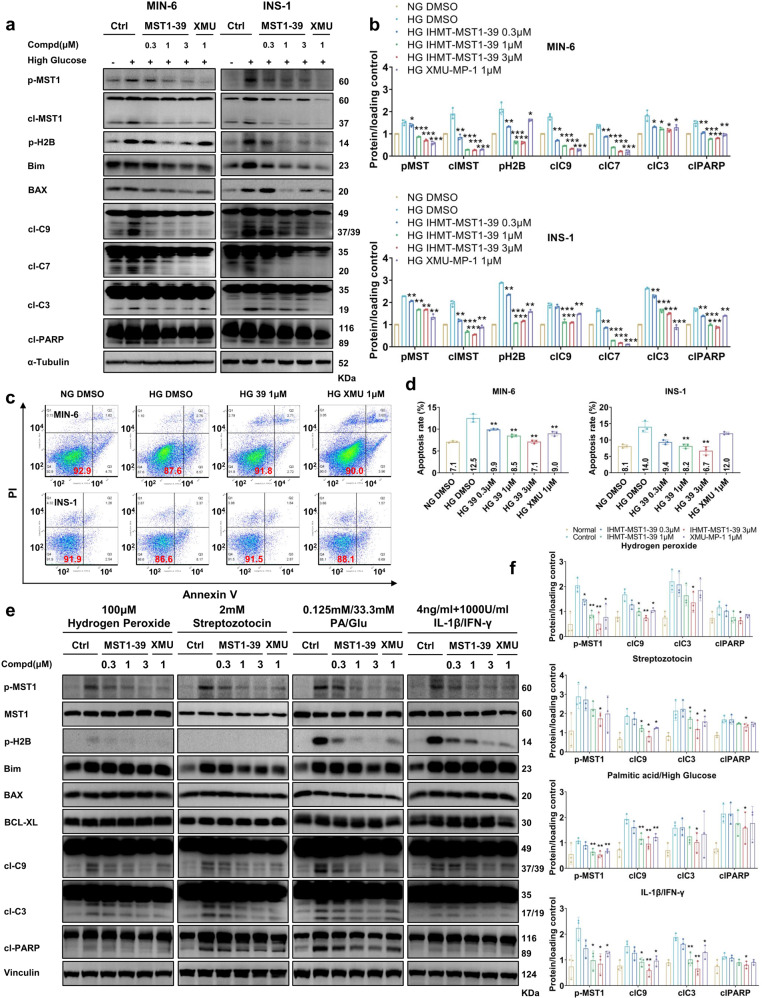
IHMT-MST1-39 blocks pancreatic β cells apoptosis through MST1 inhibition. **a**, **b** MIN-6 and INS-1 cells were exposed to high glucose (33.3 mM glucose) ±IHMT-MST1-39 and XMU-MP-1 for 72 h. Immunoblot and densitometry analysis of p-MST1, MST1, p-H2B, BIM, Caspase9, Caspase7, Caspase3 and PARP. Tubulin was used as loading control. Data were expressed ±SEM. IHMT-MST1-39 or XMU-MP-1 to normal glucose control; all by Student’s *t* tests. **c**, **d** Cell death induced by high glucose in rodent pancreas cell lines treated with IHMT-MST1-39 or XMU-MP-1 was determined by quantification of Annexin-V and PI positive cells. **e**, **f** Immunoblot and densitometry analysis of p-MST1, MST1, p-H2B, Bim, BAX, BCL-XL, Caspase9, Caspase3, PARP and Vinculin in MIN-6 cells stimulated with or without diabetogenic conditions. Data were expressed ±SEM (*n* = 3). Hydrogen peroxide (H_2_O_2_) or Streptozotocin (STZ) or Palmitic acid/High glucose (PA/HG) or Inflammatory cytokine (IL/IF) to control, *P < 0.05 IHMT-MST1-39 and XMU-MP-1 to vehicle-treated rodent pancreas cell line under the same diabetogenic conditions; *P* values determined by Student’s *t* test

Previous studies have shown that caspase3 promotes the activation of MST1 and vice-versa, both of which promote the initiation of apoptotic signaling cascade in pancreatic β cells.^10,15,16^ On the other hand, MST1 kinase activates the mitochondrial-related cell death signaling in β cells through modulating the BH3-only protein member BIM and BAX.^10^ Therefore, we investigated whether IHMT-MST1-39 was capable of inhibiting MST1-dependent apoptosis signaling within β cells in high glucose condition. At concentrations ranging from 0.3 to 3 μM, IHMT-MST1-39 reduced the phosphorylation levels of endogenous MST1 and H2B in a variety of cell lines, including MIN6, INS-1, RIN-M5F and Beta-TC/6 in a dose-dependent manner. Notably, high glucose-induced cleavage of MST1, caspase9, caspase7 and caspase3 in MST1-inhibited rodent pancreas cell lines were less than those in control cells (Fig. 2a, b and Supplementary Fig. S2a, b). Since MST1 protein is pro-apoptotic kinase, IHMT-MST1-39 treatment prevented cell death induced by high glucose, as determined by Annexin V and PI staining (Fig. 2c, d and Supplementary Fig. S2c, d). The efficacy of IHMT-MST1-39 to inhibit MST1 activation and restore β cell survival under multiple diabetogenic conditions was further confirmed using rodent pancreatic β cell line (MIN-6) with various stress conditions in vitro. As our results shown, MST1 was highly upregulated by all diabetic conditions upon chronic exposure, shown by its autophosphorylation. In contrast, IHMT-MST1-39 potently inhibited MST1 activation induced by H_2_O_2_ and STZ, and apoptosis as represented by p-H2B, caspase9, caspase3 and PARP cleavage in β cells. Again, MST1 activation and caspase-3 cleavage induced by the glucolipotoxicity at high glucose condition in combination with palmitic acid was dose-dependently abolished by IHMT-MST1-39. Similarly, caspase-3 activation induced by inflammatory cytokines (IL-1β/IFN-γ) was largely inhibited by IHMT-MST1-39 (Fig. 2e, f). Together, these data established that IHMT-MST1-39 can rescue β cell apoptosis induced by diabetogenic conditions in vitro.

### IHMT-MST1-39 improves pancreatic β cell function and survival under diabetogenic conditions

Next, we investigated the effects of the compound on β cell function and survival. At the transcription level, we found the mRNA levels of crucial genes for β cell function and survival, such as PDX1, Glut2, Insulin1, and NKX6.1, were restored in MIN6 and INS-1 cells with MST1 suppression in high-glucose condition. Meanwhile, the level of Arx1, a crucial gene for α cells, was not changed (Supplementary Fig. S3a, b). Consistently, at the protein level, we found that IHMT-MST1-39 can also restore the protein expression of crucial markers for β cells function and physiological status, including transcription factors PDX1, NKX6.1, the glucose transporter Glut2, as well as nuclear antigen Ki67 associated with cell proliferation. Similar results were seen at cellular level in INS-1 cells that were cultured at high glucose condition with palmitic acid, followed by labeling of insulin and four important markers. Under this condition, cells showed morphological changes with smaller cell size, increased number of insulin-negative cells, as well as reduced numbers of cells positive for nuclear PDX1 and NKX6.1 expression. In addition, many cells that still express insulin had lost their NKX6.1 and PDX1 expression, and the expression of Glut2 and Ki67 was also reduced. However, with IHMT-MST1-39 treatment, the pathological phenotypes were significant rescued (Supplementary Fig. S3e, f). To further confirm the effects of IHMT-MST1-39 treatment on cellular toxicity induced in various diabetogenic conditions, we assessed β cell viability using Cell Titer-Glo assay in MIN-6 and INS-1 cells. Based on pre-determined doses for MST1 activation (Supplementary Fig. S2e, f), we found a dose-dependent upregulation in MST1 autophosphorylation level after exposure to various diabetogenic conditions in MIN-6 cells, and observed improved cell survival following IHMT-MST1-39 treatment in both MIN-6 and INS-1 cells (Supplementary Fig. S3c, d). Consistently, we also observed protective effects of IHMT-MST1-39 against cellular apoptosis induced in diabetogenic conditions in primary isolated islets from murine, leporid, and human tissues (Supplementary Fig. S4a–d). These results confirmed the effects of IHMT-MST1-39 in the protection of β cell function and survival status.

### IHMT-MST1-39 reverses diabetic symptoms in the MLD-STZ mouse model of T1D

**Fig. 3:**
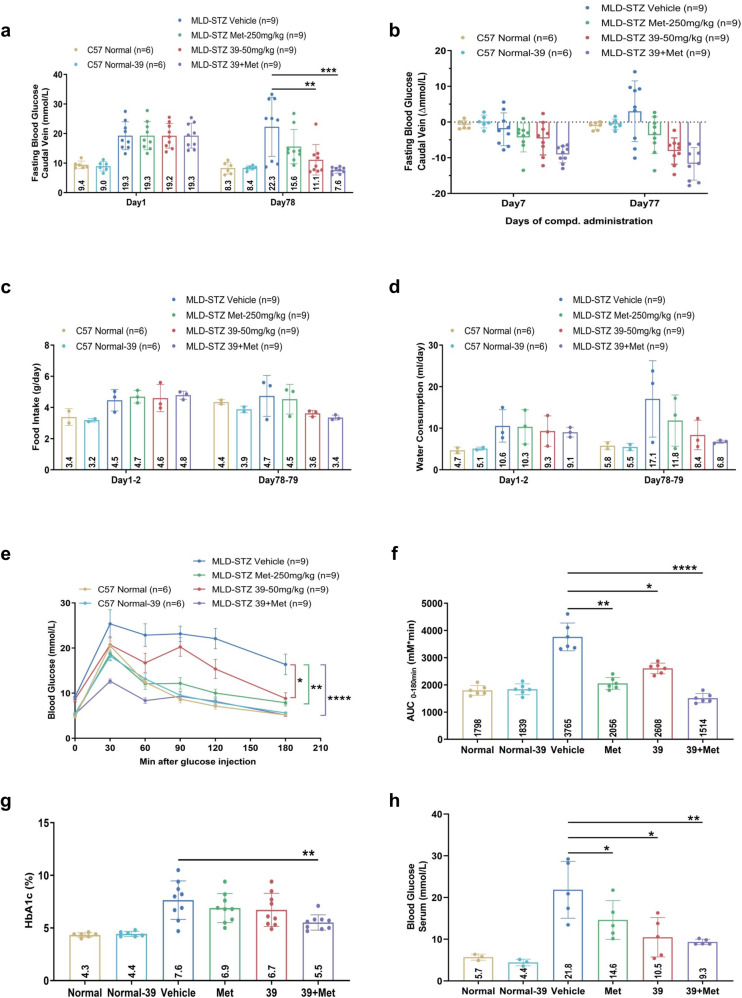
IHMT-MST1-39 reverses diabetic symptoms in the MLD-STZ mouse model of T1D. **a**–**d** Fasting blood glucose (caudal vein), food intake and water consumption measurements for once a week. **e**–**f** Intra-peritoneal glucose tolerance test (IPGTT) performed during 0 min and 180 min after glucose injection. Respective area-under-the-curve (AUC) shown in **f**. **g**, **h** HbA1c and blood glucose (serum) level was measured by Chemray-240/800. Data were expressed ±SEM. *p < 0.05, IHMT-MST1-39 compared with vehicle group mice; *P* values determined by Student’s *t* test

After confirming the protective effects of IHMT-MST1-39 on β cells, we next conducted the experiments in vivo using the MLD-STZ induced T1D mouse model to evaluate the effects of IHMT-MST1-39 treatment. MLD-STZ models induce the activation of cell apoptotic pathways and autoimmune-mediated failure of pancreatic β cells functions, which lead to progressive hyperglycemia and severely impaired glucose tolerance in WT mice.^24–26^ Firstly, we examined oral bioavailability and pharmacokinetic characterization of IHMT-MST1-39 is studies in different species (Supplementary Table S1a) and found that this compound is distributed in multiple organs with especially high accumulation in the liver and pancreas (Supplementary Table S1b). These PK properties indicated that IHMT-MST1-39 would be suitable for oral administration. To construct T1D mouse model, wild-type C57BL6/J mice were injected with STZ at a dose of 50 mg/kg per day for 5 consecutive days and then subjected to 12-weeks drug treatments starting from day 18 after the first STZ injection. It has been common practice in preclinical research and application to combine drugs throughout history, and metformin is an orally available drug wildly used in diabetic patients, so we included metformin and its combination with our IHMT-MST1-39 in drug efficacy study. Hyperglycemia was evident by day 18 after MLD-STZ treatment with blood glucose levels progressively increasing throughout the study of 12-weeks, which was accompanied with severely impaired glucose tolerance in the MLD-STZ treated control mice. However, diabetic mice treated with IHMT-MST1-39 alone and drug combination had lower levels of blood glucose (Fig. 3a, b), food intake (Fig. 3c) and water consumption (Fig. 3d) during the entire 12-weeks study, and they exhibited significantly improved glucose tolerance (Fig. 3e, f). Importantly, IHMT-MST1-39 alone had no effect on glycemia, food intake, and water consumption in non-diabetic C57BL6/J mice (Fig. 3a–d). We measured the glycosylated hemoglobin (HbA1c) and blood glucose in serum at the end of the experiments, and found dramatically elevated HbA1c and serum blood glucose in vehicle-treated MLD-STZ mice, and these levels were significantly reduced in animals treated with IHMT-MST1-39 and metformin combination (Fig. 3g, h). To recapitulate the effects of MST1 inhibition on restoration of β cell function in vivo, we examined pancreas isolated from treated mice and observed the protective effects of IHMT-MST1-39 against cellular apoptosis in pancreatic tissue from MLD-STZ mice (Supplementary Fig. S5a, b). Compared to vehicle treated samples with reduction in PDX1, NKX6.1, Glut2 and Insulin1 expression, we found that the expression levels of those crucial genes for β cell survival were restored in mice treated with IHMT-MST1-39 alone and drug combination in the MLD-STZ models (Supplementary Fig. S5c). Further, hematoxylin-and-eosin (HE) staining of the pancreatic tissue showed that the histological structure of islet was obviously destroyed in the vehicle group, and it was improved in the mono-therapy groups and the combination group (Supplementary Fig. S5d). Together, these results suggested that the remarkable glycemic control effects of IHMT-MST1-39 are mediated by β cell apoptosis reduction and function restoration in T1D mouse model.

### IHMT-MST1-39 activates AMPK signaling in an MST1-independent manner

**Fig. 4:**
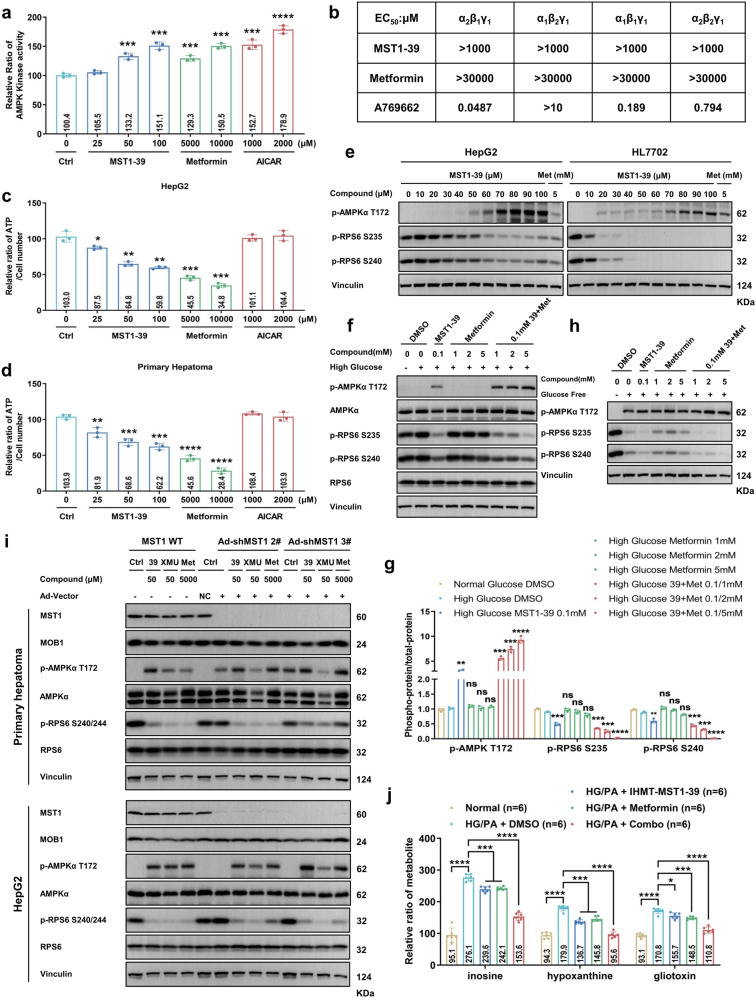
IHMT-MST1-39 activates AMPK signaling in an MST1-independent manner. **a** IHMT-MST1-39 activated AMPK kinase activity. Metformin and AICAR were positive control, 5000/10,000 μM Metformin and 1000/2000 μM AICAR were used for comparison with IHMT-MST1-39. **b** In vitro activity of IHMT-MST1-39 on recombinant AMPK heterotrimeric proteins. **c**, **d** The measurement of ATP. 24 h incubation with Metformin (5000/10,000 μM), and AICAR (1000/2000 μM) were used as control. Data were expressed ±SD. of three independent samples. **e** HepG2 and HL7702 cells were exposed to high glucose ±IHMT-MST1-39 and Metformin for 24 h. p-AMPKα, p-RPS6(S235/236), p-RPS6(S240/244) and Vinculin were analyzed by western blotting. **f**, **g** HEK293T cells were transferred into high glucose DMEM medium containing MST1-39/metformin or high-glucose DMEM containing the combination of MST1-39 and metformin, followed by cell harvest and lysate preparation after 2 h for Western blotting analysis, all treatment groups were compared to high-glucose control. **h** HEK293T cells were transferred into glucose-free DMEM medium containing MST1-39/metformin or glucose-free DMEM containing the combination of MST1-39 and metformin, followed by cell harvest and lysate preparation after 2 h for Western blotting analysis. **i** Primary hepatoma and HepG2 cells transduced with Ad-mCherry (Negative control, NC) or Ad-mCherry-shMST1 for 48 h. Cells were exposed to high glucose with compounds for 24 h. MST1, MOB1, p-AMPKα, AMPKα, p-RPS6(S240/244), RPS6 and Vinculin were analyzed by western blotting. **j** Statistical analyses of 3 metabolites in human primary hepatoma cells with High glucose (33.3 mM)/Palmitic acid (0.125 mM) (HG/PA) after treatment of compounds or DMSO for 48 h

As the most commonly used oral hypoglycemia agent worldwide,^27,28^ metformin activates AMPK by increasing cellular ratio of AMP to ATP through inhibiting mitochondrial respiration and adenosine triphosphate production.^27^ Given that AMPK is a key regulator in metabolic diseases and a potential target for the treatment of diabetes mellitus, and the above synergistic effects we saw with the combination of metformin and IHMT-MST1-39, we investigated whether IHMT-MST1-39 itself is involved in the regulation of metabolic status by activating AMPK. Using a cell-based AMPK kinase assay, we observed that AMPK activity induced by IHMT-MST1-39 was significantly increased in HepG2 cells in a dose-dependent manner (Fig. 4a). This efficacy was comparable to treatment with the currently available AMPK activator metformin. In addition, although the reported AMPK activator A769662 directly activated heterotrimeric AMPK complexes, neither metformin nor our compound showed any direct effects on AMPK activation (Fig. 4b).

AMPK directly invigilates cellular ATP level and modulates cell metabolism in response to cellular energy status.^29^ In contrast to AICAR, addition of IHMT-MST1-39 did cause changes in intracellular level of ATP in HepG2 cells, demonstrating that AMPK activation by IHMT-MST1-39 is an effect of respiratory chain impairment (Fig. 4c). We confirmed these results in human primary hepatoma cells, in which ATP level reduction was significant at 100 μM of IHMT-MST1-39 (Fig. 4d). It’s known that AMPK activation maintains the TSC1/2 activity, leading to the inactivation of Rheb-GTPase and the downregulation of mTOR signaling.^30^ Therefore, we next investigated whether IHMT-MST1-39 was able to activate AMPK in HepG2 and HL7702 cells, and observed a dose-dependent dephosphorylation of a major AMPK downstream substrate ribosomal protein S6 (RPS6) at Ser235/236 and Ser240/244 residues after 24 h treatment. A corresponding increase in Thr172 phosphorylation on the AMPK α subunit was also detected (Fig. 4e).

Given the key function of AMPK in cell metabolism modulation, we investigated whether AMPK activity is synergistically regulated by IHMT-MST1-39 combination with metformin under high-glucose and glucose-free conditions. We measured the phosphorylation level of AMPKα treated by drug combination and found that, although AMPK was not activated by high glucose condition upon chronic exposure, drug combination synergistically activated AMPK signaling in HEK-293T cells compared to starvation condition (Fig. 4f–h). Mechanistically, silencing of endogenous MST1 upregulated AMPKα activity in diabetic mice,^31^ so we further investigated whether IHMT-MST1-39 activates AMPK by inhibiting MST1 signaling. We first examined IHMT-MST1-39 and XMU-MP-1 on AMPK activation in human hepatoma cells, and found that both MST1 inhibitors were able to activate AMPK at a similar concentration (Fig. 4i). However, after MST1 silencing by adenovirus-mediated shRNA knockdown, we observed that IHMT-MST1-39 remained effective, while XMU-MP-1 showed a partially reduced effect under MST1-deficient condition (Fig. 4i). We next examined whether MST1 signaling is dependent of AMPK. We looked at MST signaling pathway in MIN-6 cells, and found that MST1 and MOB1 activation induced by the glucolipotoxicity at high-glucose condition in combination with palmitic acid was abolished by IHMT-MST1-39. Furthermore, we observed mildly increased phosphorylation level of AMPKα through IHMT-MST1-39 treatment (Supplementary Fig. S6a). Importantly, MST1 signal was not influenced by AMPK signaling either by loss of AMPK activity or direct activation, suggesting that MST1 functions independently of AMPK.

To further confirm the regulatory effects of IHMT-MST1-39 in liver metabolism, we then conducted metabolome analysis on human primary hepatoma cells cultured in the presence of high glucose and palmitic acid using liquid chromatography tandem mass spectrometry (LC-MS).^32,33^ Among the metabolites with differential abundance, 24 were found significantly different (Supplementary Fig. S6b). Previous studies have shown that, the accumulations of inosine, hypoxanthine and gliotoxin are associated with the metabolic abnormalities or diabetes.^34–37^ Our analysis showed that the levels of those three metabolites in primary hepatoma cells were abnormally increased in the HG/PA-treated control group, while these were partially rescued in the mono-therapy groups and nearly recovered to normal levels in the combination group (Fig. 4j). These data suggested that MST1 signaling inhibition is dispensable for IHMT-MST1-39 to induce AMPK activation, and our compound treatment ameliorates metabolic disorders under diabetic condition.

### IHMT-MST1-39 prevents diabetic hyperglycemia in T2D mouse models

MST1 is constitutively activated in the pancreatic islets of genetically defective diabetic mice, such as diabetic Lepr^db^ mice (db/db mice).^10^ To evaluate whether MST1 inhibition by IHMT-MST1-39 can manage hyperglycemia in T2D mouse model in vivo, obese db/db mice were treated daily with IHMT-MST1-39 or vehicle for 10 weeks. Starting from the first week, the body weights (Supplementary Fig. S7a), blood glucose levels (Supplementary Fig. S7b), food intake (Supplementary Fig. 7c) and water consumption (Supplementary Fig. S7d) did not increase significantly and remained stable in IHMT-MST1-39 treated group, while the blood glucose levels exhibited dramatic increase in vehicle-treated db/db mice over the period of 10 weeks. In addition, mice treated with IHMT-MST1-39 after 12 h fasting showed lower blood glucose levels at all time points in intra-peritoneal glucose tolerance tests (Supplementary Fig. S7e, f). Furthermore, we observed dramatically elevated glycosylated hemoglobin (HbA1c) in vehicle-treated db/db mice. On the contrary, these levels were reduced by IHMT-MST1-39 treatment (Supplementary Fig. S7g). We also confirmed the protective effective of IHMT-MST1-39 against cellular apoptosis in pancreatic tissue from db/db mice (Supplementary Fig. S8a, b). We then performed H&E staining and insulin immunolabeling on pancreatic islets from diabetic mice and found that IHMT-MST1-39-treated mice had more islets formation after 10 weeks of drug administration, whereas vehicle-treated db/db mice had no significantly restorative effect (Supplementary Fig. S8c, d). Cryo-EM analysis also revealed the anti-apoptotic effect of IHMT-MST1-39 in pancreas section from obese db/db mice (Supplementary Fig. S8e), indicating that it ameliorated diabetic hyperglycemia through the preservation of β cell survival and function.

**Fig. 5:**
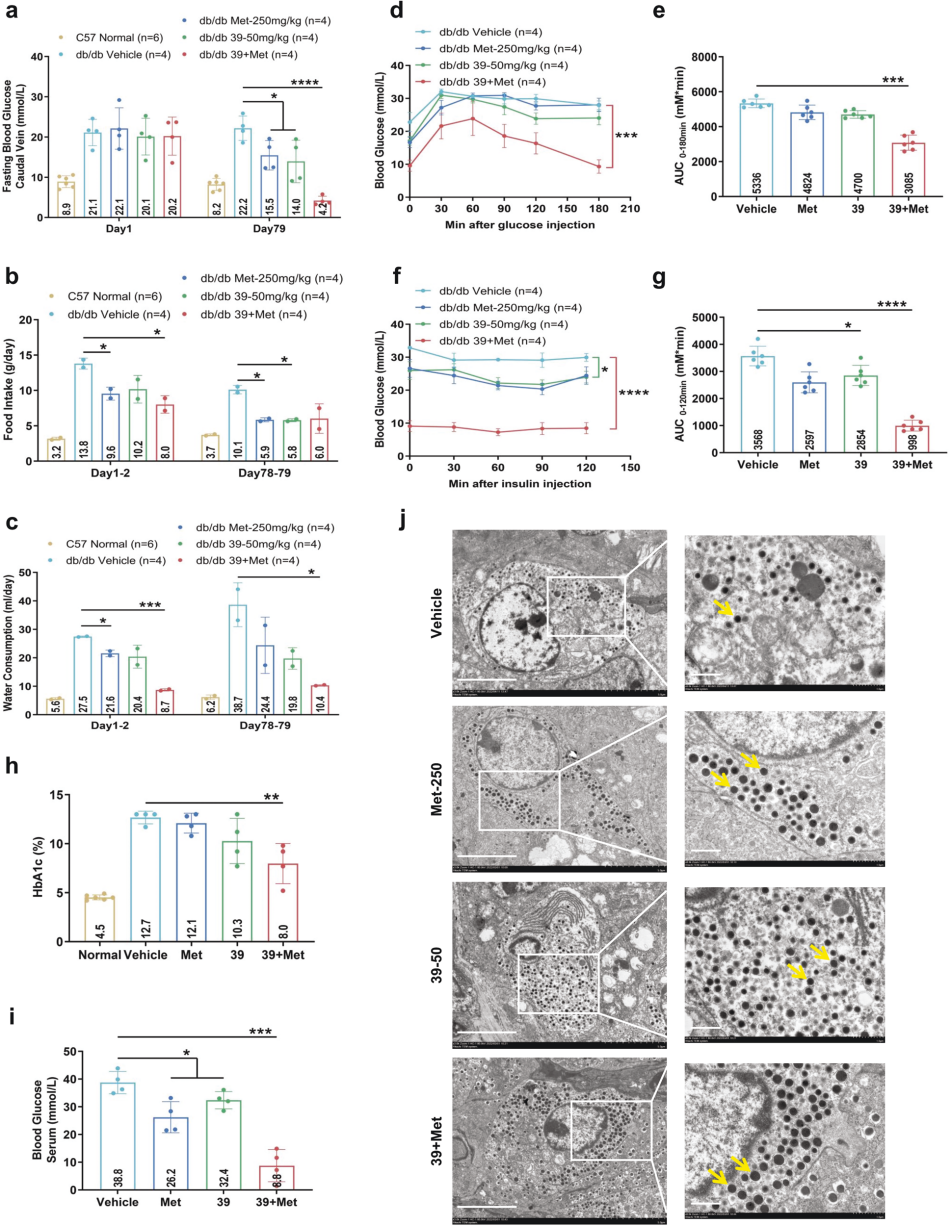
Combination of IHMT-MST1-39 and metformin prevents diabetic hyperglycemia in obese db/db mouse model of T2D. **a**–**c** Fasting blood glucose, food intake and water consumption measurements for once a week. **d**, **e** Intra-peritoneal glucose tolerance test (IPGTT) measured during 0 min and 180 min after glucose injection. Respective area-under-the-curve (AUC) shown in **e**. **f**, **g** Intra-peritoneal insulin tolerance test (IPITT) performed during 0 min and 120 min post insulin injection. Respective area-under-the-curve (AUC) shown in **g**. Data were expressed ±SEM. *p < 0.05 IHMT-MST1-39 to vehicle control; *P* values determined by Student’s *t* test. **h**, **i** HbAc1 and blood glucose (serum) level was measured by Chemray-240/800. Data were expressed ±SEM. *p < 0.05, IHMT-MST1-39 compared with vehicle group mice; *P* values determined by Student’s *t* test. **j** Isolated pancreases from db/db mice, islets activity was analyzed by cryo-sections of pancreases (as yellow arrow shows, black circles represent insulin), Scale bar, 5 μm (left), 1 μm (right)

Although IHMT-MST1-39 mono-therapy showed relatively good anti-diabetic effects in db/db mice, the blood glucose levels did not restore to normal levels. Metformin has shown beneficial effects in the management of T2D as a widely used first-line treatment for diabetes mellitus, so we next investigated whether IHMT-MST1-39 has synergistic anti-diabetic effect in combination with metformin in vivo. Obese db/db mice were then treated with IHMT-MST1-39 at 50 mg/kg/day, metformin at 250 mg/kg/day, or combined administration of these two drugs, and their FBG levels were monitored weekly during the course of the experiments. As Fig. 5a showed, the FBG level decreased in the mono-therapy groups (IHMT-MST1-39 or metformin), but the decrease was more significant in the combination group during experimental period. Also, the food intake and water consumption were improved more significantly in the combination group than the monotherapy groups relative to the vehicle group (Fig. 5b, c). Encouragingly, the FBG levels, food intake and water consumption in the combination group were restored to nearly normal levels. We also used an intraperitoneal glucose tolerance test (IPGTT) to evaluate the glucose tolerance at the 12th week in mice fasted for 12 h prior to the assay. The combination group exhibited the most significant (*p* < 0.001) anti-diabetic effect according to the blood glucose and area under the curve (AUC) data, compared to the vehicle groups (Fig. 5d, e).

Additionally, intra-peritoneal insulin tolerance test (IPITT) results also showed that T2D mice treated with the drug combination exhibited mostly improvement in insulin resistance (Fig. 5f, g). The lowest HbA1c and blood glucose in sera were observed in the combination group, indicating a synergistic anti-diabetic effect in combination therapy (Fig. 5h, i). We found that free glucose decreased dramatically and the glycogen level was significantly elevated in the liver of T2D mice treated with IHMT-MST1-39 or metformin, and most significantly in combination group (Supplementary Fig. S9a, b). Accordingly, compared to vehicle-treated liver and pancreas, higher phosphorylation levels of AMPKα and attenuated RPS6 phosphorylation were observed in tissues treated with the combination of IHMT-MST1-39 and metformin (Supplementary Fig. S9c, d). Further, hematoxylin-and-eosin (HE) staining of the pancreatic tissue showed that the histological structure of islet was destroyed in the vehicle group, and it was improved in the mono-therapy groups and more significantly in the combination group (Supplementary Fig. S9e). Cryo-EM analysis of pancreas section also showed more functional β cells and more insulin secretion in islets, indicating improved β cell survival and function in combination therapy treated db/db mice (Fig. 5j).

**Fig. 6:**
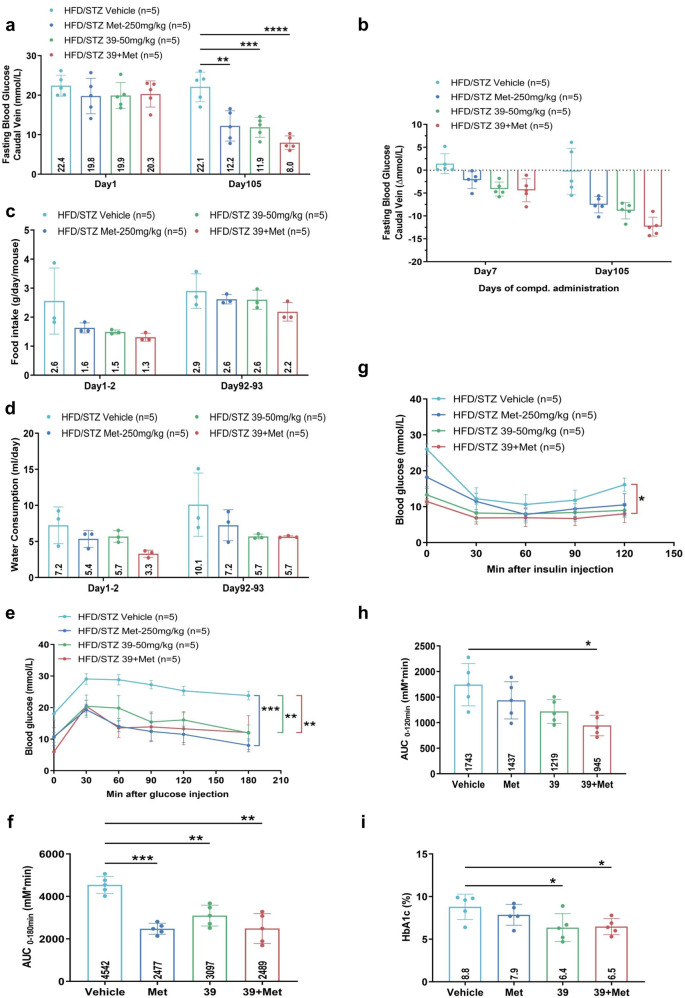
Combination of IHMT-MST1-39 and metformin decreases hyperglycemia progression in HFD/STZ-induced mouse model of T2D. **a**–**d** Fasting blood glucose (caudal vein), food intake and water consumption measurements for once a week. **e**, **f** Intra-peritoneal glucose tolerance test (IPGTT) performed during 0 min and 180 min after glucose injection. Respective area-under-the-curve (AUC) shown in **f**. **g**, **h** Intra-peritoneal insulin tolerance test (IPITT) performed during 0 min and 120 min post insulin injection. Respective area-under-the-curve (AUC) shown in **h**. **i** HbA1c level was measured by Chemray-240/800. Data were expressed ±SEM. *p < 0.05, IHMT-MST1-39 compared with vehicle group mice; *P* values determined by Student’s *t* test

We further confirmed the antidiabetic effect of combination therapy in another mouse model of T2D. The HFD-STZ induced T2D mouse model was induced by commercial high-fat diet (HFD) feeding followed by multiple low-dose STZ injections and the FBG level over 11.1 mmol/L was set as the standard for hyperglycemia. Diabetic mice were then treated with IHMT-MST1-39 at 50 mg/kg/day, metformin at 250 mg/kg/day, or combined administration of these two drugs. As expected, the FBG levels, food intake and water consumption decreased in the mono-therapy groups (IHMT-MST1-39 or metformin), but more significantly in the combination group during experimental period (Fig. 6a–d). Additionally, both glucose tolerance and insulin resistance were improved more significantly in the combination group than the monotherapy groups tested by IPGTT and IPITT (Fig. 6e–h). The lower HbA1c was observed in the combination group, indicating a better anti-diabetic effect (Fig. 6i). We also confirmed the protective effects of combination therapy against cellular apoptosis in pancreatic tissue, and the activation of AMPK signaling in liver and pancreatic tissue. Pancreatic histology further showed that the histological structure of islet was improved in the mono-therapy groups and the combination group (Supplementary Fig. S10). Altogether, our results showed that IHMT-MST1-39 and metformin synergistically prevented diabetic hyperglycemia and improved glucose homeostasis in T2D mice through simultaneous modulation of the activities of MST1 and AMPK.

## Discussion

Pharmacological manipulation of the Hippo pathway could provide therapeutic opportunity for tissue repair and regeneration. As key factors in this pathway, MST1/2 kinases play a major role in organ growth control.^38–40^ MST1 promotes apoptosis through modulation of multiple downstream targets such as LATS, histone H2B, and FOXO family members.^11,41^ This is most evident in T1D, where persistent autoimmunity leads to the destruction and consequent loss of β cells.^10^ By suppressing the MST1-mediated death signal, studies showed a similarly deleterious effect of MST1 that induces apoptosis in response to injury in autoimmune-mediated β cell destruction in the MLD-STZ mouse model, which occurs in the absence of insulin resistance. MST1 abolishment preserves β cell mass, improves β cell function, and prevents islet degeneration as indicated by maintenance of islet structure, density, and size.^10^ Although many current diabetic therapies aim to relieve the symptoms of the disease, there is still an urgent need for strategies to reduce the loss of functional pancreatic β cells. Given the important role of MST1 in β cell failure and apoptotic signaling induced by pro-apoptotic factors, therapeutic strategies aiming at blocking MST1 activity could protect β cells from autoimmune attack and preserve β cell functions. Thus, the use of pharmacological or genetic methods to inhibit MST1 could impede the progress of T1D. Consistent with previous studies,^42^ in which treatment with MST1/2 inhibitor XMU-MP-1 improves glucose tolerance in MLD-STZ induced diabetic model, we also observed significant improvement in intra-peritoneal glucose tolerance test in mice treated with IHMT-MST1-39 alone.

Metformin can activate the cellular energy sensor AMPK by inhibition of mitochondrial function. Once activated by increased ratio of AMP/ATP (indicating cellular energy balance interfered), AMPK recovers energy homeostasis through initiating the catabolic pathways, shutting down the cellular processes that consume ATP.^43^ More importantly, a recent study reported that inhibition of AMPK activity aggravated apoptosis, impaired mitochondrial function, and increased the ROS formation during hypoxia and reoxygenation in H9C2 cells.^44^ More importantly, AMPK could be activated when MST1 was deficient or inhibited. Based on the studies above, we believe that it is important to investigate the effect of MST1 inhibition in T2D. In support of our hypothesis, two independent studies showed potential protective effects to β cells upon MST1 inhibition. In the first study, a multi-target kinase inhibitor, neratinib, was shown to exhibit both glycemia control and β cell protection effects in rodent diabetic models, which the authors believed is through MST1 inhibition by this drug.^45^ In a recent case report, the effects of MST1 inhibition were further implicated in a breast cancer patient with T2D during the treatment with neratinib, which ameliorated the hyperglycemia in the patient.^46^

Our studies using multiple T2D mouse models showed that, IHMT-MST1-39 could markedly improve β cell survival and function, which could be of potential therapeutic value in diabetic patients. In accord with numerous reports, we also observed that the beneficial effects of metformin in T2D mouse models, whereas the combination of IHMT-MST1-39 and metformin had significantly cooperative effects in vivo with, improvements in multiple aspects. Especially, it becomes clear that IHMT-MST1-39 alone and drug combination treatment prevented the increase in glycemia over time (Fig. 6a, b). However, cellular apoptotic analysis of pancreatic tissues did not show highly significant difference between the mice treated with IHMT-MST1-39 alone and those treated with drug combination (Supplementary Fig. S10a, b). We hypothesize that the result might be caused by unknown mechanisms, which improved metabolic status in liver through synergistically enhancing the activity of AMPK. Taken together, our present study demonstrates that restoration of pancreatic β cell survival and improvement of metabolic status using the combination of IHMT-MST1-39 and metformin may contribute to the elevated anti-diabetic effect in vivo.

It is well known that inhibition of the core components in Hippo pathway leads to the upregulation of YAP/TAZ activity, which is associated with increased cell growth and proliferation. Previous observations with XMU-MP-1 and TT-10, two inhibitors of Hippo pathway, confirmed that these compounds positively regulate cell propagation in hepatocytes^47^ and cardiomyocytes,^48^ which suggest potential carcinogenic effects of these compounds with prolonged use. However, healthy mice treated with IHMT-MST1-39 (50 mg/kg) daily for consecutive 11 weeks did not exhibit adverse effects in glycemia, food intake, water consumption, and cancerous growth (Fig. 3). In addition, assessment of organ index, serum markers of liver function, and histological analysis of pancreas confirmed no significant injury in these organs by IHMT-MST1-39 treatment (Supplementary Fig. S11). Thus, IHMT-MST1-39 appears to be safe as a targeted therapeutic agent to treat diabetes mellitus.

Pharmacokinetic studies in different species including mice, rats and dogs following intravenous (iv) injection and oral administration showed that IHMT-MST1-39 has a good drug-like PK profile including suitable half-life and stable exposure. IHMT-MST1-39 possesses acceptable bioavailability in mice (F = 129.6%), rats (F = 52.6%), and dogs (F = 151%), which indicated that it is suitable for oral administration (Supplementary Table S1a). We also evaluated the tissue distribution of IHMT-MST1-39 using a solvent system of 5% DMSO and 25% Solutol HS-15. It exhibited good drug exposure in both liver and pancreas, which are the primary targeted tissues in diabetes models (Supplementary Table S1b). In conclusion, we developed a potent and selective MST1 inhibitor, IHMT-MST1-39, which exhibits enhanced anti-apoptosis effect in ex vivo rodent pancreatic β cell lines under multiple diabetogenic conditions. Our findings added more evidence that pharmacological manipulation of MST1 signaling might provide protective benefit for pancreatic β cells upon complicated apoptotic stresses. Based on our results above, IHMT-MST1-39 would be not only a good pharmacological tool to investigate the MST1-mediated physiology and pathology, but also a potential drug candidate for the treatment of diabetes. The encouraging anti-diabetic effects of combination therapy in vivo also suggested that simultaneous pharmacological inhibition of MST1 and activation of AMPK represents a novel promising approach for diabetes therapy.

## Methods

### Animal study

All procedures and experimental protocols were approved by the animal care regulations of Hefei Institutes of Physical Science, Chinese Academy of Sciences (DWLL-2019-05).

The male Sprague–Dawley rats (190–210 g) were provided by Laboratory Animal Center of Anhui Medical University (Hefei, China). C57BL/6 J (5w, male) mice, ICR mice (8w, male) and db/db mice (7w, male) were purchased from Gem-Pharma tech Co., Ltd. (Nanjing, China). These animals were housed in an air-conditioned room (22~24 °C and 50~60% humidity) with a 12 h light/dark cycle, and were allowed free access to food and water in agreement with US National Institutes of Health animal care guidelines. The animals were used in experiments after one-week adaptation.

## Supplementary information

Supplementary Materials-SIGTRANS-07602R1

## Data Availability

The data that support the findings of this study are available from the lead corresponding author upon reasonable request.
