# Supplementary material for: Discovery of IHMT-MST1-39 as a novel MST1 kinase inhibitor and AMPK activator for the treatment of diabetes mellitus
Source: Signal Transduct Target Ther. 2023 Apr 5;8:143. doi: 10.1038/s41392-023-01352-4 (PMC10073293; doi:10.1038/s41392-023-01352-4)
Supplement: Supplementary file 1 — Supplementary Materials-SIGTRANS-07602R1 [file 41392_2023_1352_MOESM1_ESM.docx]

Supplementary Materials for

**Discovery of IHMT-MST1-39 as a Novel MST1 Kinase Inhibitor and AMPK Activator for the Treatment of** [**Diabetes**](file:///E:\%E8%BD%AF%E4%BB%B6%E5%AE%89%E8%A3%85\%E6%AC%A7%E9%99%86%E8%AF%8D%E5%85%B8\Dict\7.5.2.0\resultui\dict\?keyword=diabetes)**Mellitus**

Junjie Wang^1,2,5^, Ziping Qi^1,3,5^, Yun Wu^1,3,5^, Aoli Wang^1,3,5^, Qingwang Liu^1,3^, Fengming Zou^1,3^, Beilei Wang^1,3^, Shuang Qi^1,3^, Jiangyan Cao^1,2^, Chen Hu^1,3^, Chenliang Shi^1,2^, Qianmao Liang^1,2^, Li Wang^1,3^, Jing Liu^1,3*^, Wenchao wang^1,3*^, Qingsong Liu^1,2,3,4*^

1. Anhui Province Key Laboratory of Medical Physics and Technology, Institute of Health and Medical Technology, Hefei Institutes of Physical Science, Chinese Academy of Sciences, Hefei, Anhui 230031, P. R. China
2. University of Science and Technology of China, Hefei, Anhui 230026, P. R. China
3. Hefei Cancer Hospital, Chinese Academy of Sciences, Hefei, Anhui 230031, P. R. China
4. Precision Medicine Research Laboratory of Anhui Province, Hefei, Anhui 230088, P. R. China
5. These authors contribute equally

**Corresponding Authors**

* Qingsong Liu (E-mail: [qsliu97@hmfl.ac.cn](mailto:qsliu97@hmfl.ac.cn))

* Wenchao Wang (Email: [wwcbox@hmfl.ac.cn](mailto:wwcbox@hmfl.ac.cn))

* Jing Liu (E-mail: [jingliu@hmfl.ac.cn](mailto:jingliu@hmfl.ac.cn))

**This PDF file includes:**

Materials and Methods.

Figures. S1 to S14.

Tables S1

**Materials and Methods**

**Reagents and antibodies**

IHMT-MST1-39 was synthesized in the lab with the procedure provided in the Fig. S12-S14. XMU-MP-1, metformin, AICAR, SBI-0206965 and A769662 were purchased from Med Chem-Express Inc (Shanghai, China). The following commercial antibodies were used: phospho-MST1 (Thr183) (#49332), MST1 (#3682), phospho-LATS1 (Thr1079) (#8654), LATS1 (#3477), [phospho-MOB1 (Thr35)](https://www.cellsignal.cn/products/primary-antibodies/phospho-mob1-thr35-d2f10-rabbit-mab/8699?site-search-type=Products&N=4294956287&Ntt=mob1&fromPage=plp) (#8699), MOB1 (#13730), phospho-Histone H2B (#6959), Histone H2B (#12364), Caspase-9 (#9504), Caspase-7 (#12827), Caspase-3 (#14220), PARP (#9542), Bim (#2933), BAX (#2772), Bcl-2 (#3498), BCL-XL (#2764), phospho-AKT (Ser473) (#4060), phospho-AKT (Thr308) (#13038), phospho-p44/42 (Erk1/2) (Thr202/Tyr204) (#4370), PDX1 (#5679), NKX6.1 (#54551), phospho-AMPKα (Thr172) (#2535), AMPKα (#2532), phospho-S6 Ribosomal protein (Ser235/236) (#2211), phospho-S6 Ribosomal protein (Ser240/244) (#5364), S6 Ribosomal protein (#2217), Vinculin (#13901) (Cell Signaling technology). Insulin1 (67284-1-Ig), Ki67 (27309-1-AP), Glut2 (20436-1-AP) (Protein-tech). α-Tubulin (sc-8035) (Santa Cruz). GAPDH (HC301-01) (Trans-Gen Biotech). β-actin (30101ES60) (Yeasen). Anti-Flag M2 (F3165) (Sigma). Secondary antibodies (anti-rabbit IgG) (#7074). (anti-mouse IgG) (#7076) conjugated with horseradish peroxidase (Cell Signaling technology). Goat Anti-Mouse IgG(H+L) CoraLite488 conjugate (SA00013-1), Donkey Anti-Rabbit IgG(H+L) CoraLite594 conjugate (SA00013-8) (Protein-tech). All the primary and secondary antibodies were used at a dilution of 1:1000, 1:3000 for immunoblotting, respectively.

**Cell culture and islet isolation**

Islets from C57BL6/J mice (Nanjing Experimental Center, Jiangsu, China) and Yellow-rabbits (Yihua Agriculture and Animal Husbandry Technology Inc, Anhui, China) were perfused with a Liberase TM solution according to the manufacturer’s instructions and digested at 37°C, followed by washing and handpicking. INS-1, MIN-6 and RIN-M5F was cultured in complete RPMI-1640 medium (Corning, USA) at 10 mM glucose supplemented with 10% FBS. Beta-TC/6 was cultured in DMEM medium (Corning, USA) at 22 mM glucose supplemented with 15% FBS. All media included glutamate, penicillin-streptomycin and FBS (all from EXCELL Bio). INS-1 medium was supplemented with 0.05 mM β-mercaptoethanol.

**Biochemical assay of kinase activity**

The ADP-Glo kinase assay (Promega, USA) was used to determine the inhibition of MST1/2/3/4 wt by IHMT-MST1-39 and XMU-MP-1. MST1/2/3/4 wt (12.5 ng/μL) was incubated for 1 h at room temperature with substrates poly (4:1 Glu, Tyr) peptide (1.2 μg/μL) (Promega, USA) and 100μM ATP (Promega, USA) in 20 μL reaction buffer containing the serially diluted compounds or DMSO. The reaction was stopped by the addition of the ADP-Glo reagent and samples were incubated for 40 min at room temperature. Then, the kinase detection reagent was added, and the samples were incubated for 30 min to produce a luminescence signal. The detection was performed with an automated plate reader (Envision, PE, USA) and dose-response curves were fitted using Prism 8.0 (GraphPad Software, CA, USA).

**TEAD reporter assay**

TEAD-pcDNA3.1 or empty vector was co-transfected with the luciferase-pcDNA3.1 plasmid into MDA-MB-231 cells for 24 h. Then, the transfected cells were seeded at a density of 10^5^ cells/well on 6-well plates and were kept in the 5% CO2 incubator at 37°C for 12 h. On the next day, the cells were cultured in Dulbecoo’s modified Eagle’s medium (DMEM) without fetal bovine serum (FBS) for 12 h. The treatments (DMSO as control, 0.3 μM, 1 μM, 3 μM, of IHMT-MST1-39 and 1μM of XMU-MP-1) were added glucose to final concentration 66.6 mM. Cells were collected and lysed after different time points of incubation. The luciferase signal was then measured by the Bright-Glo reagent (Promega) according to the manufacturer’s recommendations with a plate reader ENVISION and normalized to the ratio in control cells incubated with DMSO.

**Cytotoxicity Cell Titer-Glo® assay**

Islets and all cell lines were transferred to a micro-plate at 100 μl per well, grown in 96-well culture plates for 12 h. Then, various concentrations of the compounds were mixed in a dose-dependent manner in 96-well micro-plates at 10000 cells/well in 100 μL of complete grow medium. Cell proliferation was measured after 24 h of treatment with the compound, 18 μL of Cell titer-Glo® reagent (Promega, USA) was added to each well. Cell viability was determined using the Cell Titer-Glo (Promega, USA). Luminescence was determined using a multimode Envision plate reader (PerkinElmer, USA) in the Cell Titer-Glo assay. Data were normalized to control (DMSO).

**Real-Time Quantitative PCR**

One microgram of total RNA from the cells or pancreas tissue was reverse transcribed with oligo dT and reverse transcriptase (Trans-Gen Biotech). Real-time quantitative PCR was performed using a Takara SYBR Green Super-mix kit and the Light-Cycler® 96 SW system (Roche). All runs were accompanied by the internal control α-Tubulin gene. The samples were run in triplicate and normalized to α-Tubulin using a threshold-based algorithm, to provide arbitrary units representing relative expression levels. Each α-Tubulin is representative of at least three independent experiments.

**PCR Primer used for real time PCR**

Insulin1: CCATCAGCAAGCAGGTCATTG

TGTGTAGAAGAAGCCACGCTCC

PDX1: AAATCCACCAAAGCTCACGC

GGTCAAGTTCAACATCACTGCC

NKX6.1: CTTCTGGCCCGGAGTGATG

GGGTCTGGTGTGTTTTCTCTTC

Glut2: TGCTGCTGGATAAATTCGCC

TCAGCAACCATGAACCAAGG

MafA: AGGAGGAGGTCATCCGACTG

CTTCTCGCTCTCCAGAATGTG

Glucagon: GAGGAACCGGAACAACATTGC

GCAATGAATTCCTTTGCTGCC

Arx1: GGCCGGAGTGCAAGAGTAAAT

TCGATGCAGTAGGAGGAGAGC

MafB: AGGACCTGTACTGGATGGC

CACTACGGAAGCCGTCGAAG

**Primary cells treatments**

Primary human hepatoma cell assays were provided by Hefei PreceDo pharmaceuticals Co.Ltd. (Hefei, Anhui, CN) and primary human islets were obtained from Blue-F Bio Inc (Shanghai, China). All studies performed with human specimens were performed with approval from Hefei Institutes of Physical Science, Chinese Academy of Sciences (Hefei, China). Ethical approval and informed consent were obtained for the use of human samples.

**Adenovirus transduction**

The high-titer adenovirus, Ad-mCherry-hMST1-shRNA expressing mCherry and human MST1 shRNA were obtained from Hanbio (Shanghai, China). Ad-mCherry-shRNA was used as control. For transduction, human primary hepatoma or HepG2 cells were plated for 24 h; then infected at a multiplicity of infection for 4 h in medium. After 4 h incubation, human primary hepatoma or HepG2 cells were washed with medium and incubated for an additional 48 h. Knockdown efficiency was evaluated by Western blot using specific MST1 antibody.

shRNA used for knockdown MST1 isoforms:

shMST1-2#: TCGAGGGCAATCTTCATGATTCCTACAAATTTCAAGAGAATTTGTAGGAATCATGAAGATTGCCTTTTTTA

shMST1-3#: TCGAGGCCAATACTATGATTGAGCACGATGATTCAAGAGATCATCGTGCTCAATCATAGTATTGGTTTTTTA

**SDS-PAGE and Immunoblot analysis**

Tissues and all cell lines were lysed with RIPA buffer and 0.1% SDS supplemented with protease and phosphatase inhibitors. Lysates were centrifuged at 13000 rpm. for 10 min at 4°C. Protein concentrations were determined with the BCA protein assay. Electrophoresis was performed on SDS-polyacrylamide gels. Proteins were separated by SDS-PAGE, were transferred onto a PVDF membrane and then were identified by immunoblot analysis with the appropriate primary antibodies at a dilution of 1:1000. HRP–conjugated antibody to rabbit IgG or to mouse IgG (1:3000 dilution for each) were from Cell Signaling technology. The protein bands were visualized with a Super-Signal Western Kit according to the manufacturer’s instructions (Millipore).

**AMPK kinase assay**

AMPK kinase activity in total cell lysates was performed using CycLex® AMPK Kinase Assay Kit (MBL, Japan), according to the manufacturer’s instructions.

A. Treatment of Cells

1.Plate adherent cells in 6 well-plate at 60-70 % confluency (around 2.5ⅹ10^5^ cells/well).

2.Incubate the culture dish at 37°C for 12 hours in CO_2_ incubator.

3.Change the medium to FCS-free, low glucose medium. Incubate at 37°C overnight in CO_2_ incubator.

4.Add appropriate amount of test compound or equivalent and vehicle for test compound to each well.

5.Incubate the culture dish at 37°C for appropriate time.

B. Cell Extraction

1. Wash cells three times with ice-cold PBS. Remove any remaining PBS by decanting. Invert the plate and blot it against clean paper towels.

2. Lyse the cells by adding 0.1 ml of Cell Lysis Buffer for 60 minutes at 4°C, with rotating at 300 rpm by an orbital microplate shaker.

3. Transfer the cell lysates to microcentrifuge tubes and centrifuge at 14000 rpm for 10 minutes at 4°C.

4.Transfer the clear lysates to new 96-well microplate or clean microcentrifuge tubes. 10 µL of these cell lysates are ready for assay.

C. Standard Assay

1. Remove the appropriate number of microtiter wells from the foil pouch and place them into the well holder. Return any unused wells to the foil pouch, refold, seal with tape and store at 4°C.

2. Prepare all samples.

3. Begin the kinase reaction by addition of 90 µL of Kinase Reaction buffer per well, cover with plate sealer, and incubate at 30°C for 30 minutes.

4. Stop the reaction by flicking out the contents.

5. Wash wells five times with Wash Buffer making sure each well is filled completely. Remove residual Wash Buffer by gentle tapping or aspiration.

6. Pipette 100 µL of Anti-Phospho-mouse IRS-1 S789 Monoclonal Antibody into each well, cover with plate sealer or lid, and incubate at room temperature for 30 minutes.

7. Wash wells five times as same as in step 5.

**AMPK activity assay**

1. Add 12 μL compound to 384-well dilution plate.

2. Dilute compound 1:3 in succession in DMSO for each column for 10 pts.

3. Transfer 0.05 μL diluted compound solution in each row to 384 assay plate using Echo, each column containing 3 replicates.

4. Add 2.5 μL enzyme working solution to 384-well assay plate, centrifuge 1000 rpm for 1 min.

5. Incubate at 25℃ for 15 min.

6. Add 2.5 μL substrate (STK-S1) working solution to initiate reaction.

7. Add 5 μL dection (XL-665 & STK-Ab) working solution to initiate reaction.

8. Incubate at 25℃ for 60 min.

9. Reading 337/665 nm and 337/620 nm fluorescence signals with BMG (Ratio 665/620).

Sources of AMPK heterotrimeric proteins and related reagents:


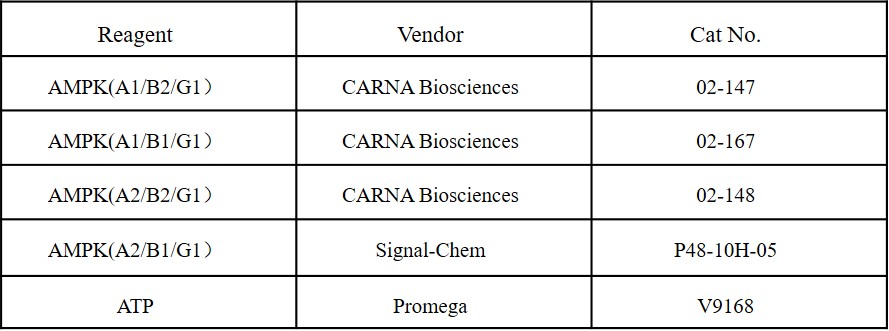


**Immunofluorescent staining**

For preparation of samples, the cells were washed three times with PBS and were fixed for 30 min with 4% paraformaldehyde at room temperature, after which additional immunofluorescence staining was applied. The primary antibodies anti-Insulin (1:200 dilution), anti-PDX1 (1:200 dilution), anti-NKX6.1 (1:400 dilution), anti-Ki67 (1:200 dilution) and anti-Glut2 (1:200 dilution) were used. Fixed cells were rinsed with PBS and then incubated for 30 min on ice with 0.3% TritonX-100 in PBS. Following permeabilization, nonspecific binding in the cells was blocked by incubation for 1 h at room temperature with 0.3% Triton X-100 and 5% BSA in PBS and cells were incubated for 1 h with specific primary antibodies (identified above). After three washes with PBS, the cells were incubated for another 1 h with secondary antibodies (Goat Anti-Mouse IgG(H+L) CoraLite488 conjugate, Donkey Anti-Rabbit IgG(H+L) CoraLite594 conjugate, 1:500 dilution). Subsequently, the cells were washed three times with PBS and were mounted with DAPI solution. All images were collected with a confocal microscope (Olympus SpinSR10).

**Diabetes models**

For MLD-STZ induced T1D model, six-week-old mice were given an intraperitoneal injection of 40 mg/kg STZ in 0.01 M citrate buffer, pH 4.2 for 5 consecutive days. Two weeks later, fasting blood glucose (FBG) level ≥ 16.7 mmol/L was set as the standard for successfully established T1D model. For HFD-STZ induced T2D model, six-week-old mice were given high food diet (HFD, 60 kcal% Fat, Dowsentec Inc., China) for eight weeks to create hyperlipemia model. Then these mice were further induced by an intraperitoneal injection of 45 mg/kg STZ in 0.01 M citrate buffer, pH 4.2 for 3 consecutive days. FBG level ≥ 11.1 mmol/L was set as the standard for successfully established T2D model. Db/db mice were spontaneous T2D model. The successfully established diabetic mice were selected and randomly assigned. IHMT-MST1-39 (50 mg/kg/day) or vehicle was delivered daily in a solvent (5% DMSO/0.5% mesylate/20% solutol HS-15 (BASF, Germany) in ddH_2_O) by oral gavage. Metformin (250 mg/kg/day) was delivered daily in an HKI solution (5% Methocellulose/0.4% Tween80 in ddH_2_O) by oral gavage. Tail venous blood samples were collected for the measurement of FBG (mice were fasted 8 h in T1D and 14 h in T2D). Throughout the whole study, food intake and water consumption were measured weekly.

**Measurement of FBG, IPGTT and IPITT**

Mice were fasted for 12 h with free access to water and tail vein blood samples were collected for the measurement of fasting blood glucose (FBG). For i.p.glucose tolerance tests (IPGTT) of control and hyperglycemic mice, mice were fasted 12 h overnight and injected with glucose at a dose of 1.5 mg/kg body weight. Blood samples were obtained at time points 0, 30, 60, 90, 120, 150 and 180 min for glucose measurements by using a Glucometer (Freestyle; Roche). For i.p.insulin tolerance tests (IPITT), mice were injected with 0.75 U/kg body weight recombinant human insulin after a 4-5 h fast, and glucose concentration was measured at time points of 0, 30, 60, 90 and 120 min post insulin administration. Glucose contents were calculated as the area under curves (AUCs).

**Pharmacokinetic Study**

Following our previously reported protocol^1^, the pharmacokinetic parameters were analyzed through non-compartment model using Win-Nonlin 6.1 software (Pharsight Corporation, Mountain View, USA), including half-life (t1/2), plasma concentration at 0 min (C0), the peak of the plasma concentration (C-max), the time to peak of the plasma concentration (T-max), the area under the plasma concentration–time curve during the period of observation (AUC0–t), the area under the plasma concentration–time curve from zero to infinity (AUC0−∞), clearance (CL), apparent volume of distribution (Vd) and the mean residence time (MRT). The oral bioavailability (F) is calculated according to the following equation: F=AUC0−∞ (oral) / AUC0−∞ (iv) × Dose (iv) / Dose (oral) ×100%.

**HE staining and immunohistochemistry**

Samples were dissected and fixed overnight in 4% neutral-buffered formaldehyde and then were dehydrated in increasing concentrations of isopropyl alcohol, followed by clearing of alcohol by xylene. The specimens were subsequently embedded in paraffin wax in cassettes for facilitation of tissue sectioning. Standard staining with hematoxylin and eosin (HE) was performed on 5 μm thickness sections from each specimen block. For immunohistochemical analysis of insulin, Pancreas sections were deparaffinized and incubated in citrate buffer at 95 °C for 40 min for antigen retrieval and then blocked with 5% horse serum for 30 min followed by incubating 24 h at 4°C with the primary antibodies including anti-pro-insulin (R&D; MAB13361) and anti-insulin (Abcam; #7842) antibodies (all at a dilution of 1:100). After three washes, tissue sections were incubated with Goat Anti-Guinea pig IgG (Abcam; #97154) (1:200 dilution) at room temperature for 1h and then washed three times followed with streptavidin–horseradish peroxidase conjugates (Vector Laboratories) incubation for 1h. After three washes with PBS, the slides were incubated with DAB solution (Vector Laboratories). Microscopic images were taken using a Nikon Eclipse E600 equipped with a Nikon Digital Sight DS-U1 unit (Spach Optics Inc, USA).

**Measurement of Glycogen and Free glucose**

Glycogen and free glucose content of liver sections was performed using Glycogen Microplate Assay Kit (Absin, Shanghai, China, Cat# abs580128) and Tissue Glucose Content Assay Kit (Applygen, Beijing, China), respectively, according to the manufacturer’s instructions.

**Metabolomic sequencing**

Primary human hepatoma cells metabolomic profiling was performed at the Broad Institute on the Broad Metabolomics platform^2,3^ liquid chromatography mass-spectrometry (LC-MS) were used to profile the following sets of metabolites.

**Statistical analyses**

All statistical analysis was performed using GraphPad prism v8.0. Each mice experiment was performed for mouse tissues and at least 3 independent mice were included in the analyses, as reported in all figure legends. Data were expressed ± SEM. Student’s t-test and two-way ANOVA test were used to assess statistical significance. The quantification of immunoblots, number of islets and area of islets labeling count were tabulated graphically with error bars corresponding to means±SEM and compared using two-tailed Student’s t-tests. Blood glucose changes in the diabetic models with the different treatment arms were compared across the time course of study using two-tailed paired-samples Student’s t test. Differences with a value of p < 0.05 were considered to be significant, whereas those with p < 0.001 were deemed to be highly significant.

## Figure S1


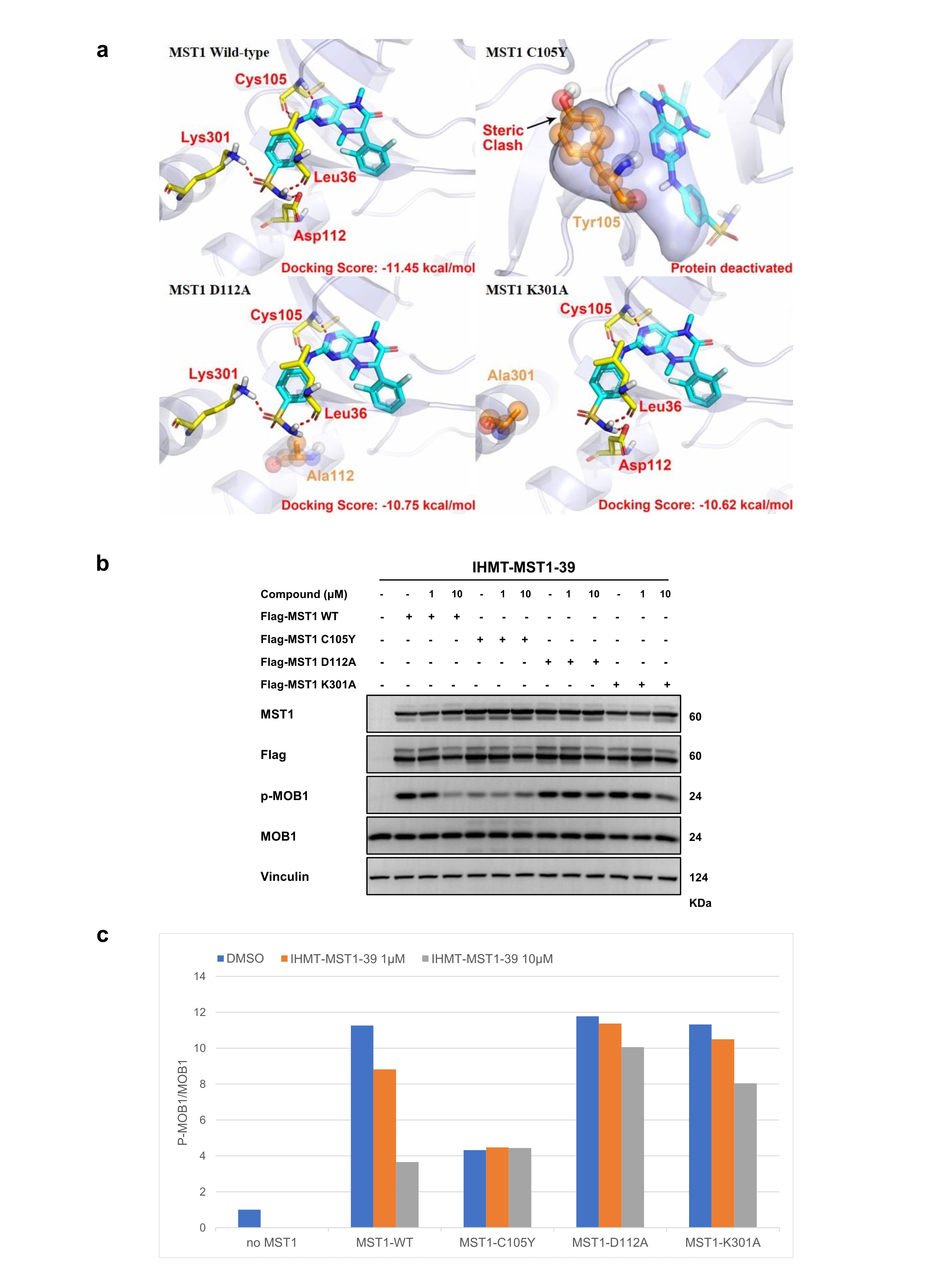


**Supplementary Figure S1** **a** Molecular modeling analyses of the binding mode of MST1 wild-type and mutants with IHMT-MST1-39. MST1 homology model based on template PDB code:6yat. **b-c** Immunoblots analysis of MST1, Flag, p-MOB1, MOB1 and Vinculin and quantification of relative levels of p-MOB1 in lysates of HEK-293T cells expressing Flag-tagged wild-type (WT) MST1 or mutant MST1 [C105Y, D112A and K301A] as indicated followed by treatment with DMSO or various concentrations of IHMT-MST1-39 (0, 1, 10 μM).

## Figure S2


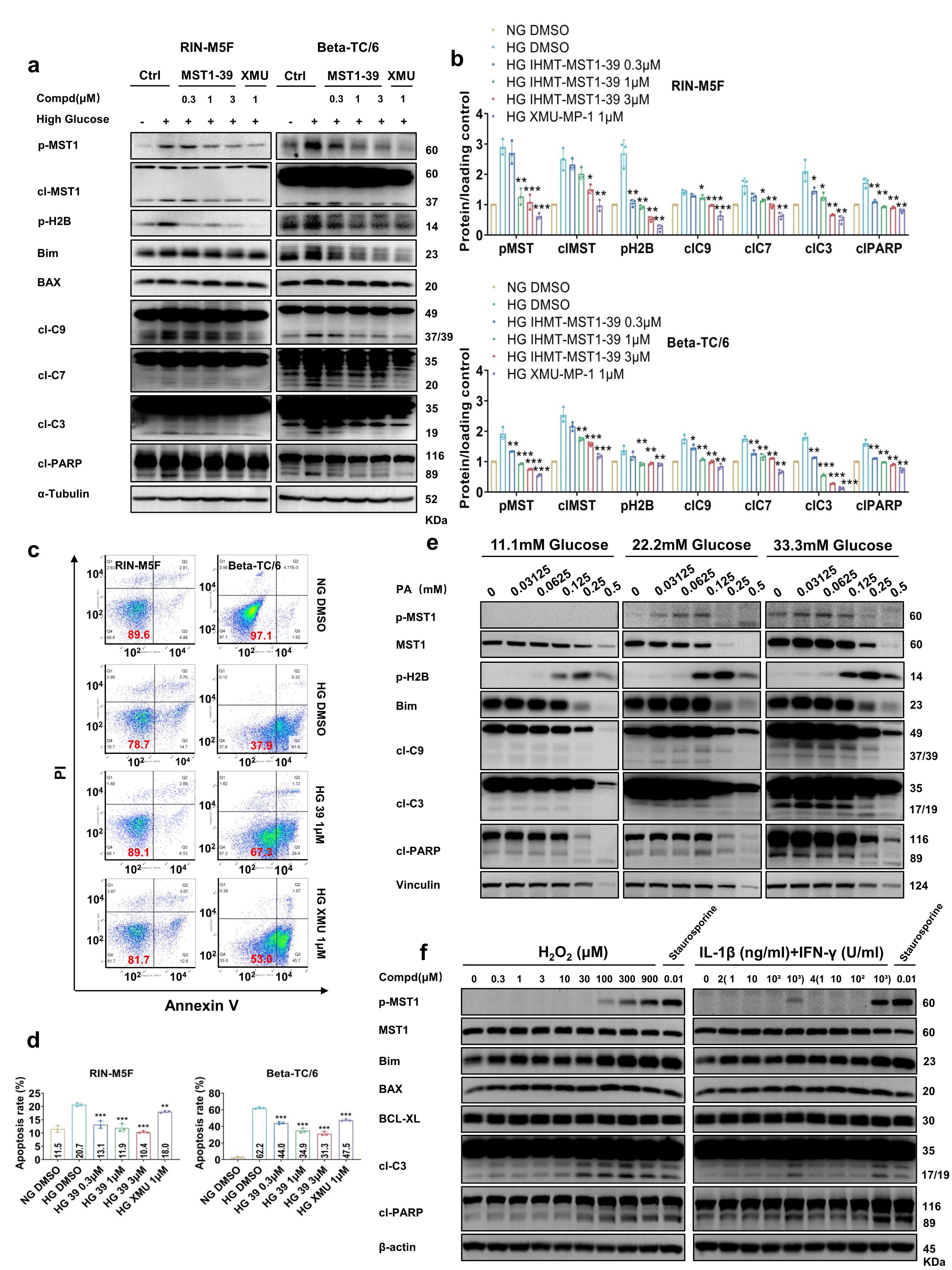


**Supplementary Figure S2** **a-b** RIN-M5F and Beta-TC/6 cells were exposed to high glucose (33.3 mM glucose) ± IHMT-MST1-39 and XMU-MP-1 for 72 h. Immunoblot and densitometry analysis of p-MST1, MST1, p-H2B, BIM, Caspase9, Caspase7, Caspase3 and PARP. Tubulin was used as loading control. Data were expressed ± SEM. IHMT-MST1-39 or XMU-MP-1 to normal glucose control; all by Student’s t tests. **c-d** Cell death induced by high glucose in rodent pancreas cell lines treated with IHMT-MST1-39 or XMU-MP-1 was determined by quantification of Annexin-V and PI positive cells. **e-f** Immunoblot analysis of p-MST1, MST1, p-H2B, BIM, BAX, BCL-XL, Caspase9, Caspase3, PARP and Vinculin in MIN-6 cells stimulated with or without diabetogenic conditions. Palmitic acid/High glucose (PA/HG) or Hydrogen peroxide (H_2_O_2_) or[Inflammatory cytokine](javascript:;) (IL/IF) to control.

## Figure S3


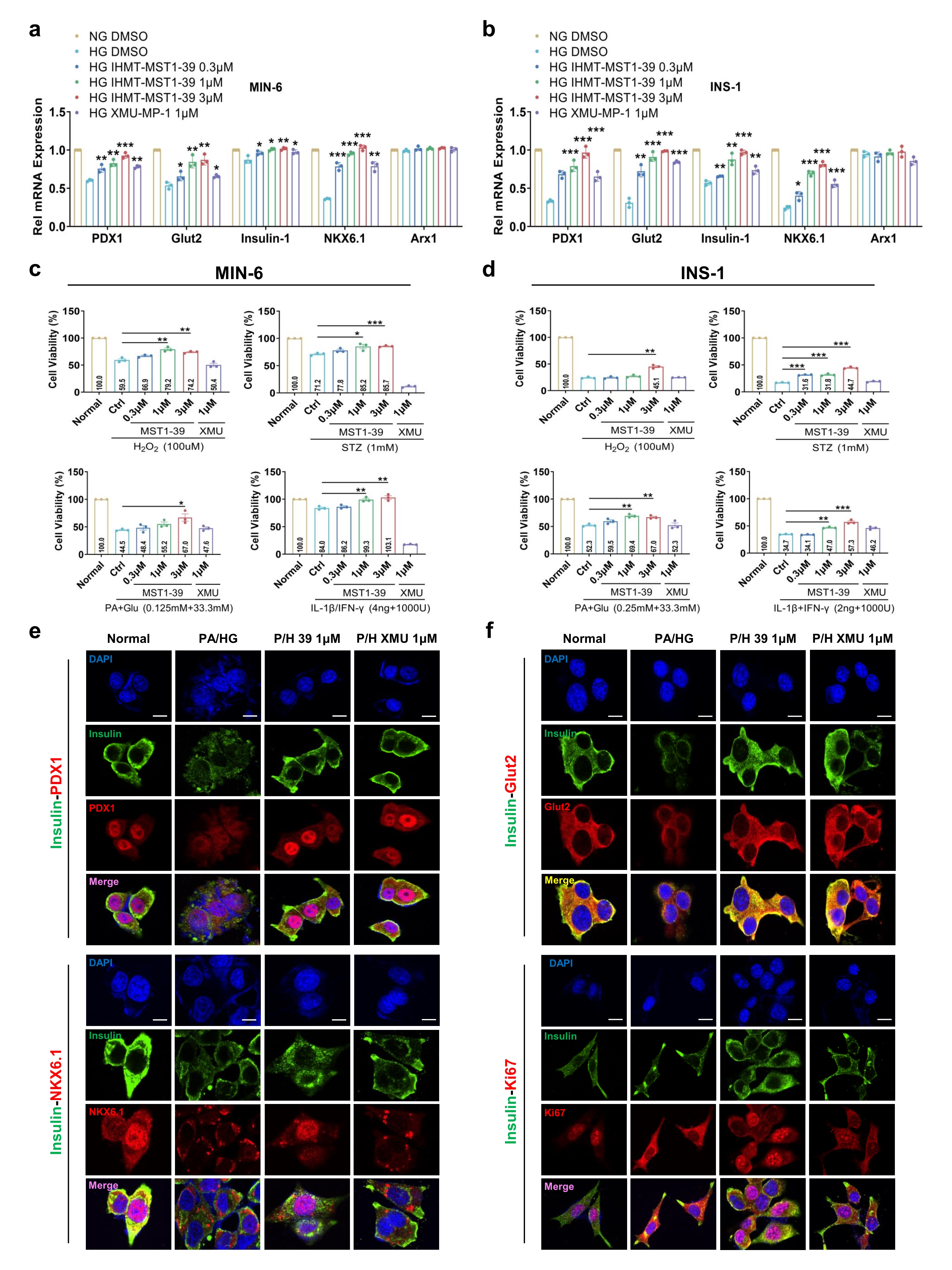


**Supplementary Figure S3 a-b** qPCR for PDX1, Glut2, Insulin1, NKX6.1 and Arx1 in rodent pancreas cell lines normalized to tubulin shown as relative changes from normal glucose control. **c-d** Cell viability assay analysis of cell survival in MIN-6 and INS-1 cells stimulated with or without diabetogenic conditions. Treatment with 0.3 μΜ,1 M and 3 μM of IHMT-MST1-39 or 1μM XMU-MP-1 for 24 h (n = 3) in each group. *p < 0.05. **e-f** Confocal microscopy of protein distribution in INS-1 cells exposed to palmitic acid/high glucose (PA/HG) after IHMT-MST1-39 or XMU-MP-1 treatment for 72 h. β cells function was analyzed by double staining of insulin (green), PDX1 (red), NKX6.1 (red), Glut2(red) and Ki67(red). Nuclei were counterstained with DAPI. Scale bars, 20 μm.

## Figure S4


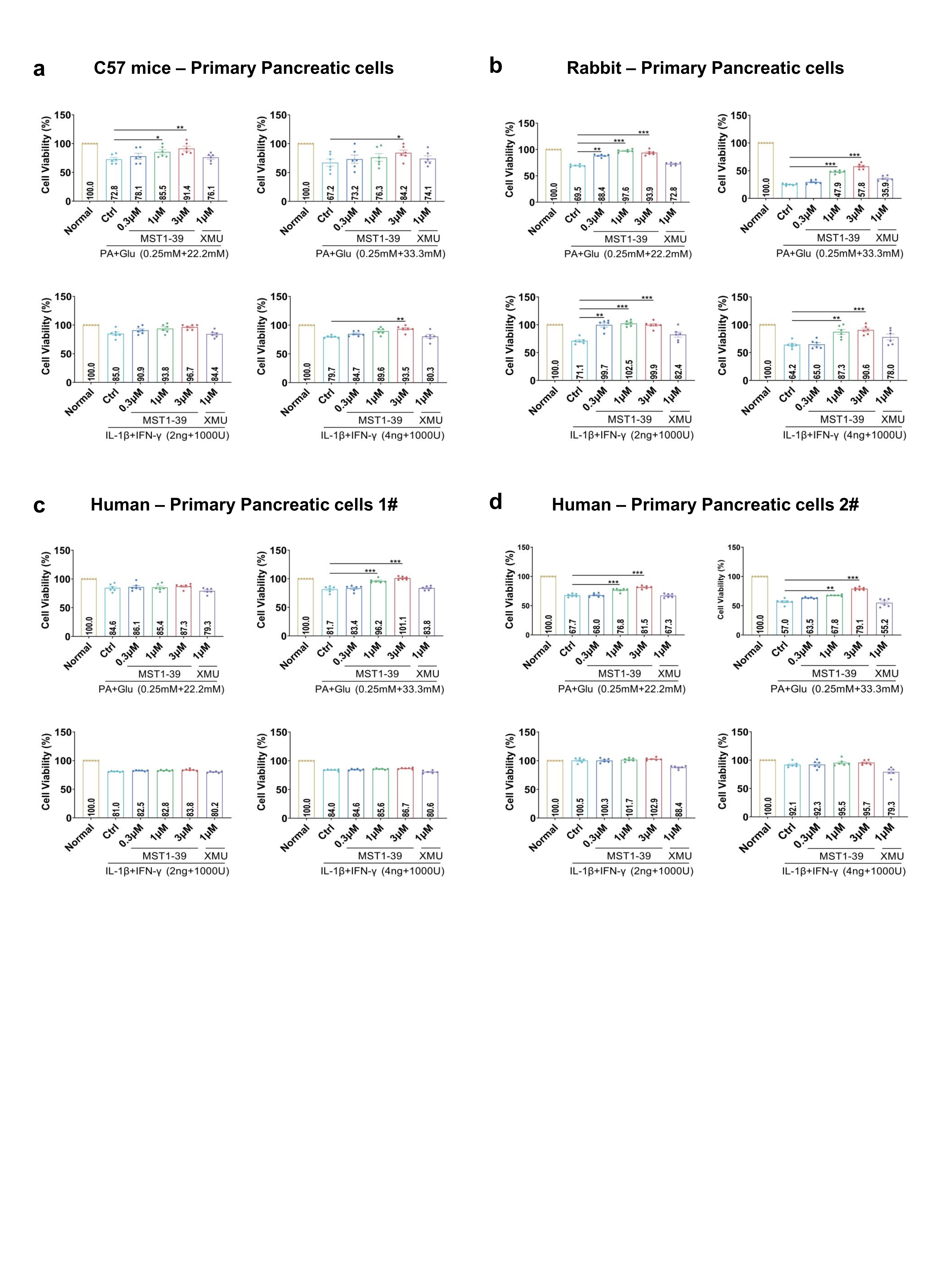


**Supplementary Figure S4 a-d** Cell viability assay analysis of cell survival in murine, leporid, and human primary pancreatic islet cells stimulated with or without diabetogenic conditions. Treatment with 0.3 μM，1 μM and 3 μM of IHMT-MST1-39 or 1μM XMU-MP-1 for 24 h (n = 6) in each group. *p < 0.05.

## Figure S5


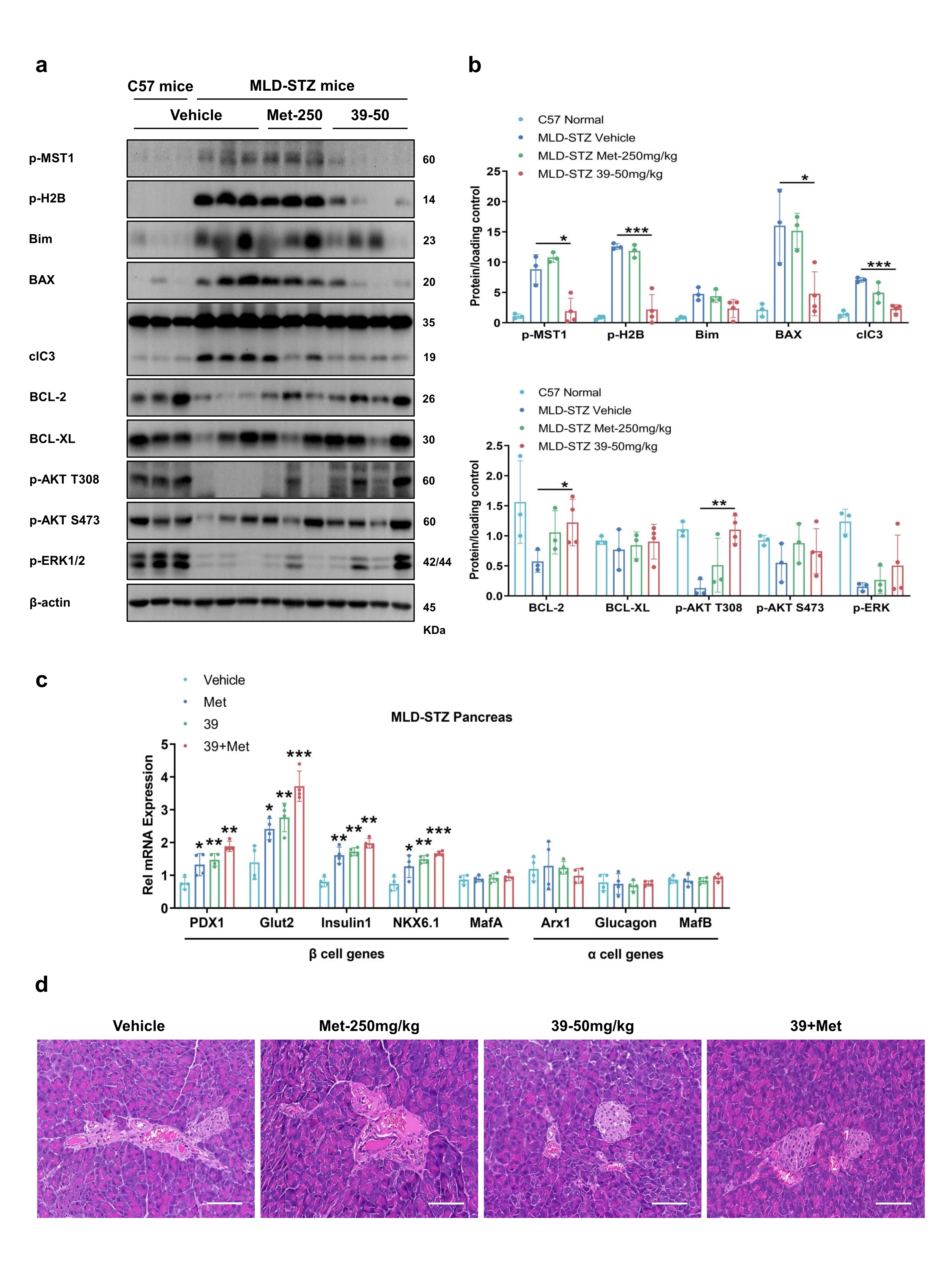


**Supplementary Figure S5 a-b** Isolated pancreases from MLD-STZ mice, Immunoblot and densitometry analysis of the apoptosis level of pancreas tissue sections in all groups of mice. C57 mice vehicle group (n = 3), MLD-STZ mice vehicle group (n = 3), MLD-STZ mice Metformin group (n = 3), MLD-STZ mice IHMT-MST1-39 group (n = 4). Data were expressed ± SEM. *p < 0.05, compound treated group compared with vehicle group mice; **p < 0.05, compound treated group compared with vehicle group mice; ***p < 0.001, compound treated group compared with vehicle group mice; P values determined by Student’s t test. **c** qPCR for PDX1, Glut2, Insulin1, NKX6.1, MafA, Arx1, Glucagon and MafB in MLD-STZ mice pancreatic tissue sections normalized to tubulin shown as change from vehicle group mice. **d** Histological analysis of pancreatic tissue sections, islets number and area were analyzed by HE stained pancreatic tissue sections (pink), Scale bar = 100 μm.

## Figure S6


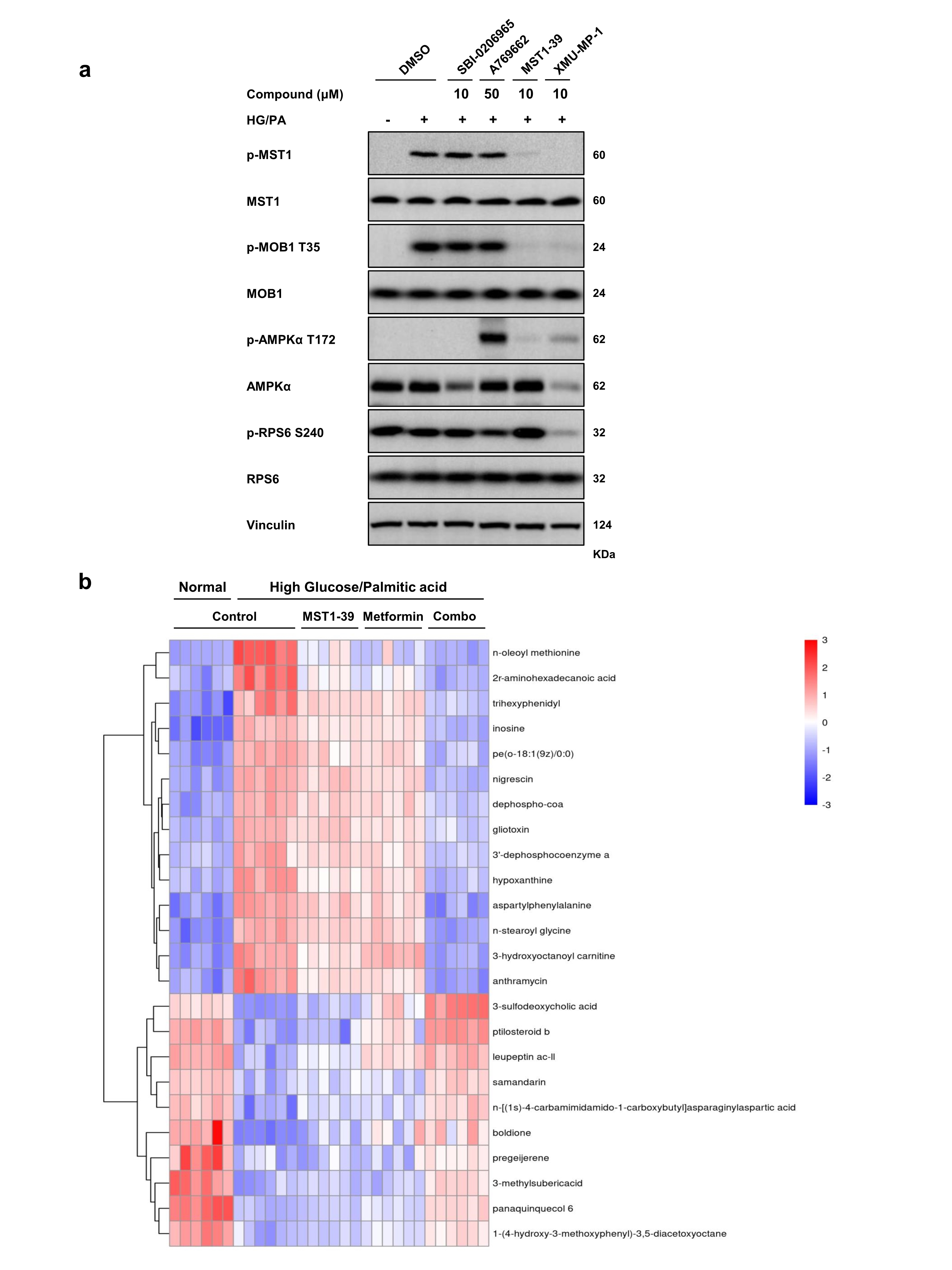


**Supplementary Figure S6** **a** MIN-6 cells were exposed to high glucose/palmitic acid (HG/PA) with compounds for 72 h. p-MST1, MST1, p-MOB1, MOB1, p-AMPKα, AMPKα, p-RPS6, RPS6 and Vinculin were analyzed by western blotting. **b** Heatmap of 24 metabolites with significantly differential abundance in human primary hepatoma cells cultured in the presence of high glucose and palmitic acid.

## Figure S7


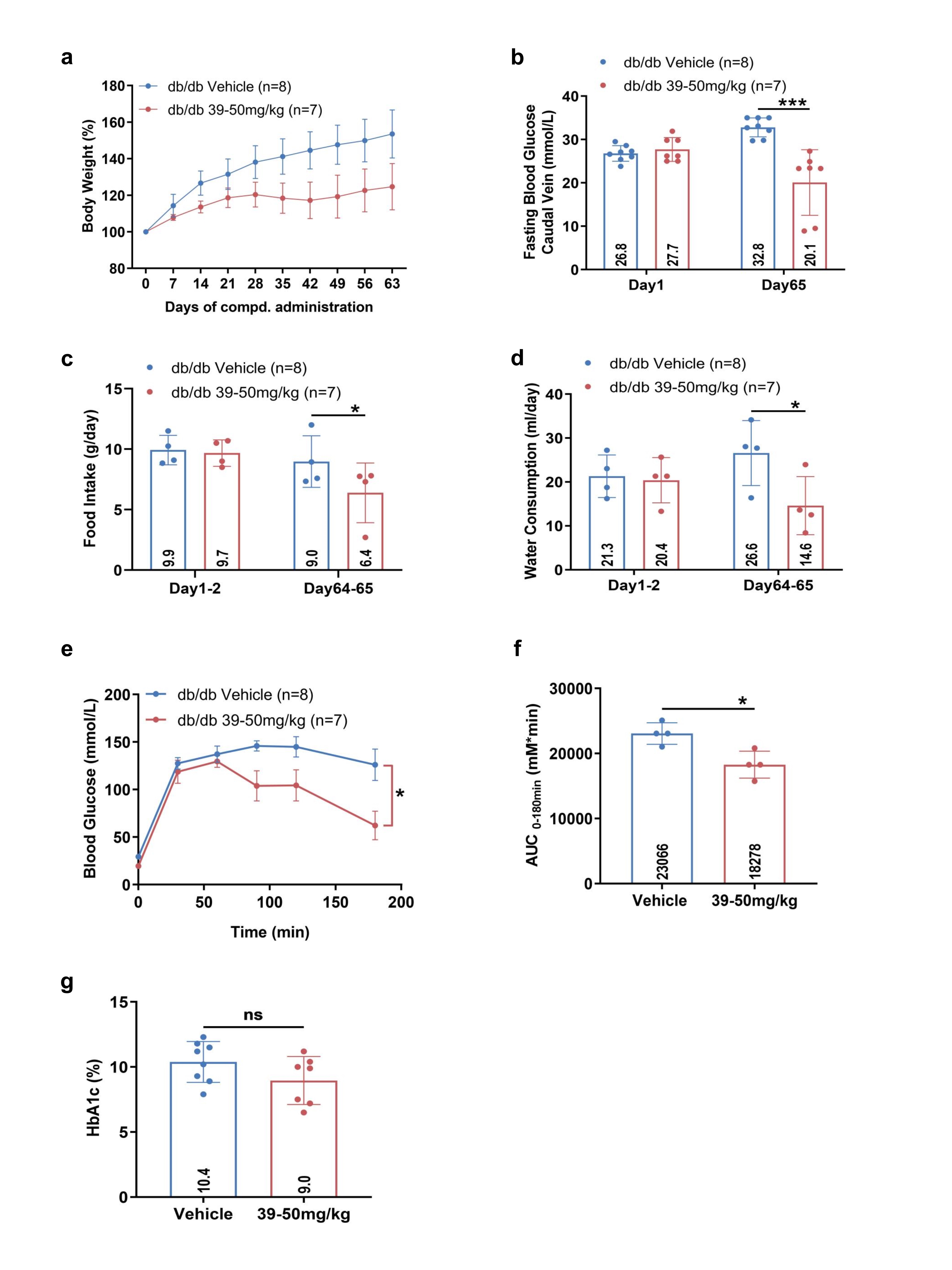


**Supplementary Figure S7** Obese diabetic Lepr (db/db) mice were randomized in two groups at the age of 6 weeks, and then, IHMT-MST1-39 was daily oral gavage at a concentration of 50 mg/kg throughout the experiment of 10 weeks. **a-d** Body weight, fasting blood glucose, food intake and water consumption measurements after IHMT-MST1-39 oral gavage for once a week. **e-f** Intra-peritoneal glucose tolerance test (IPGTT) measured during 0 min and 180 min after glucose injection. Respective area-under-the-curve (AUC) shown in f. **g** HbA1c level was measured by Chemray-240/800. Data were expressed ± SEM. *p < 0.05, compound treated group compared with vehicle group mice; **p < 0.05, compound treated group compared with vehicle group mice; ***p < 0.001, compound treated group compared with vehicle group mice; P values determined by Student’s t test.

## Figure S8


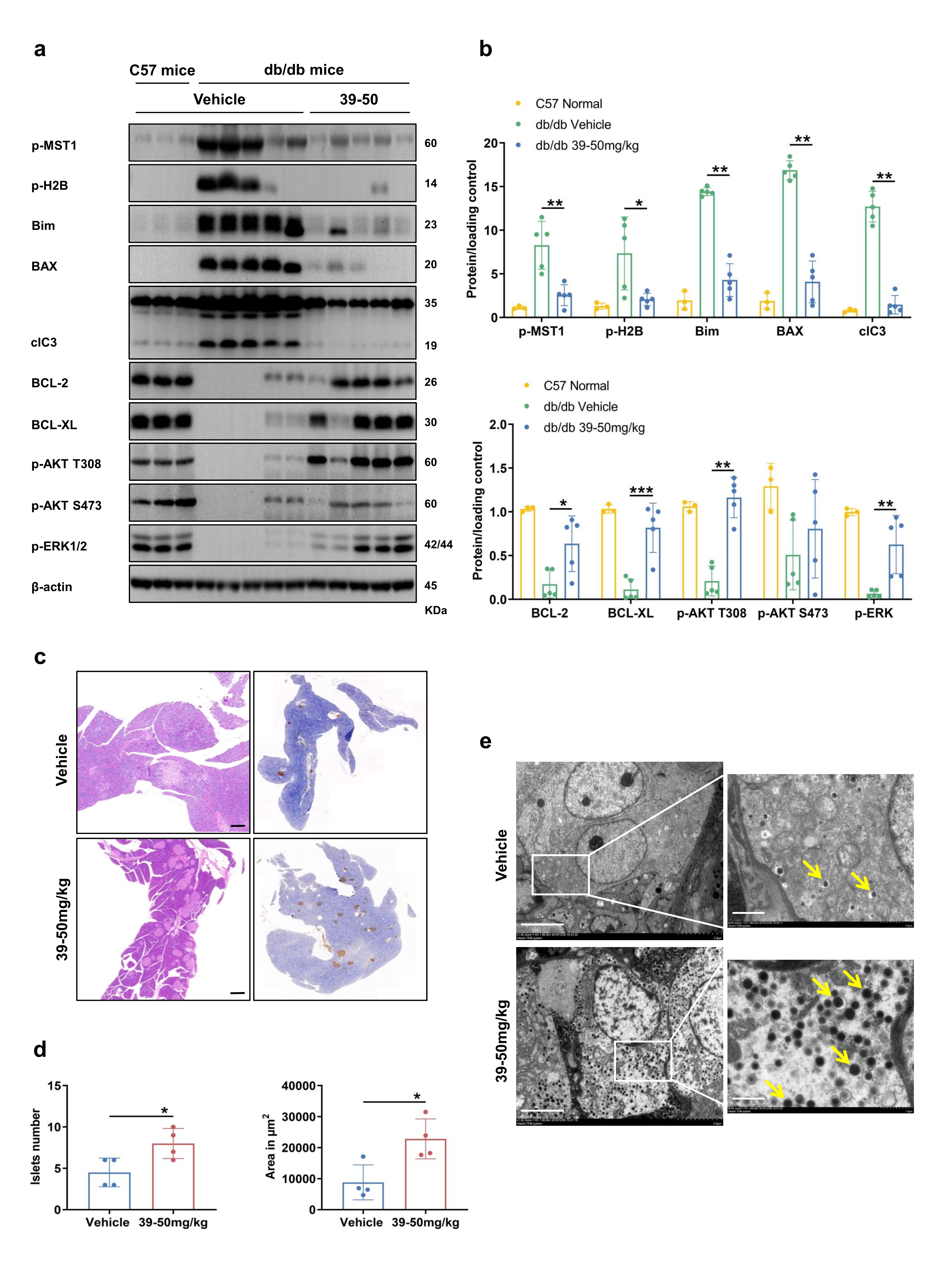


**Supplementary Figure S8** **a-b** Isolated pancreases from db/db mice, Immunoblot and densitometry analysis of the apoptosis level of pancreas tissues in all groups of mice. C57 mice vehicle group (n = 3), db/db mice vehicle group (n = 5), db/db mice IHMT-MST1-39 group (n = 5). Data were expressed ± SEM. *p < 0.05, compound treated group compared with vehicle group mice; **p < 0.05, compound treated group compared with vehicle group mice; ***p < 0.001, compound treated group compared with vehicle group mice; P values determined by Student’s t test. **c-d** Histological analysis of pancreatic tissue sections, islets number and area were analyzed by HE and IHC stained pancreatic tissue sections (pink and brownness, respectively), Scale bar = 1000 μm. **e** Isolated pancreases from db/db mice, islets activity was analyzed by cryo-sections of pancreases (as yellow arrow shows, black circles represent insulin), Scale bar, 5 μm (left), 1 μm (right).

## Figure S9


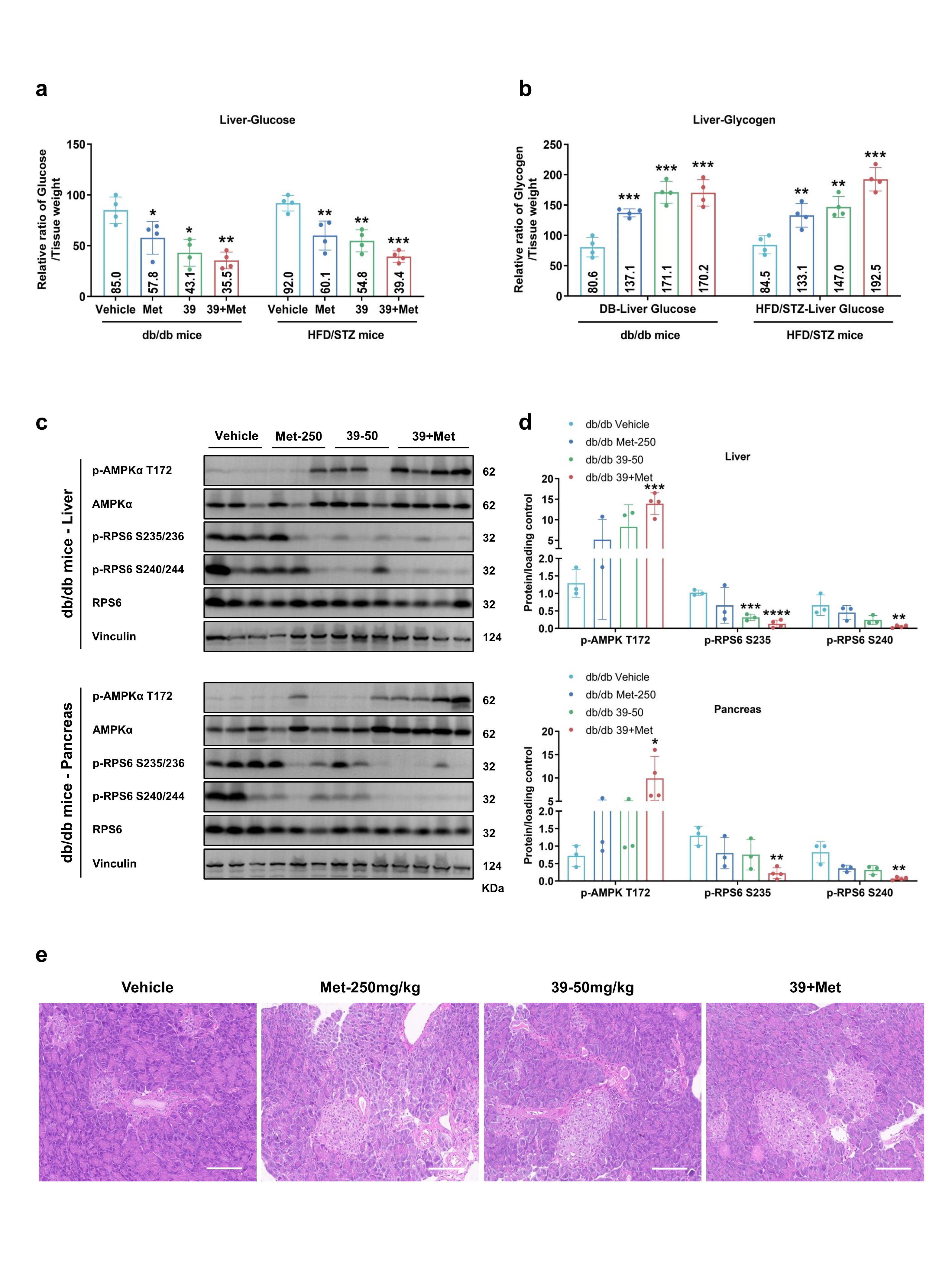


**Supplementary Figure S9** **a-b** Isolated liver and pancreases from T2D mice, the measurement of free glucose and glycogen in mice liver tissues normalized to protein content shown as change from vehicle group mice. **c-d** Immunoblot and densitometry analysis these tissues in all groups of mice. db/db mice vehicle group (n = 3), db/db mice Metformin group (n = 3), db/db mice IHMT-MST1-39 group (n = 3), db/db mice combo group (n = 4). Data were expressed ± SEM. *p < 0.05, compound treated group compared with vehicle group mice; **p < 0.05, compound treated group compared with vehicle group mice; ***p < 0.001, compound treated group compared with vehicle group mice; P values determined by Student’s t test. **e** Histological analysis of pancreatic tissue sections, islets number and area were analyzed by HE stained pancreatic tissue sections (pink), Scale bar = 100 μm.

## Figure S10


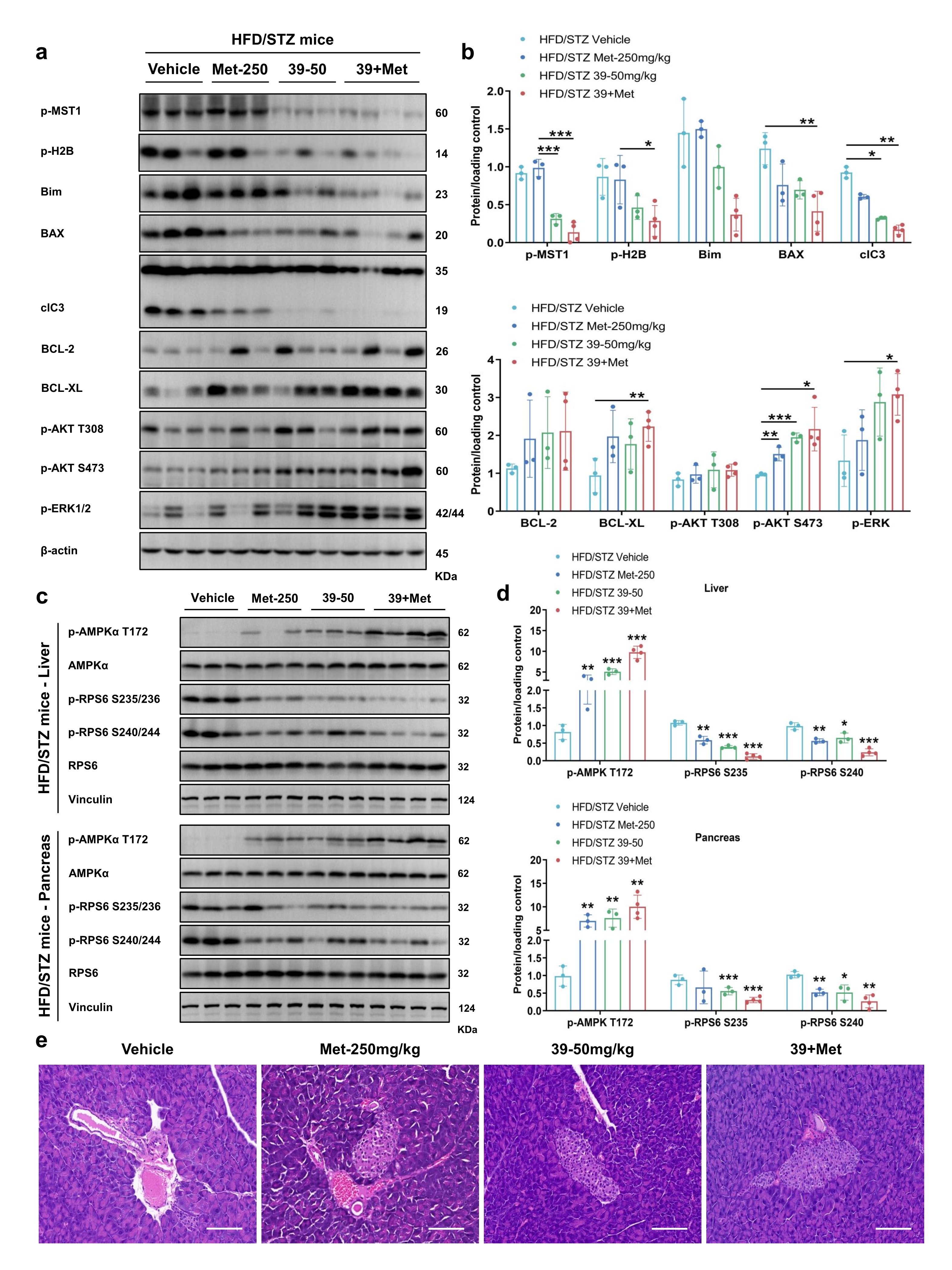


**Supplementary Figure S10** **a-b** Isolated liver and pancreases from HFD/STZ mice, Immunoblot and densitometry analysis of the apoptosis level of pancreas tissues in all groups of mice. HFD/STZ mice vehicle group (n = 3), HFD/STZ mice Metformin group (n = 3), HFD/STZ mice IHMT-MST1-39 group (n = 3), HFD/STZ mice combo group (n = 4). **c-d** Immunoblot and densitometry analysis these tissues in all groups of mice. HFD/STZ mice vehicle group (n = 3), HFD/STZ mice Metformin group (n = 3), HFD/STZ mice IHMT-MST1-39 group (n = 3), HFD/STZ mice combo group (n = 4). Data were expressed ± SEM. *p < 0.05, compound treated group compared with vehicle group mice; **p < 0.05, compound treated group compared with vehicle group mice; ***p < 0.001, compound treated group compared with vehicle group mice; P values determined by Student’s t test. **e** Histological analysis of pancreatic tissue sections, islets number and area were analyzed by HE stained pancreatic tissue sections (pink), Scale bar = 100 μm.

## Figure S11


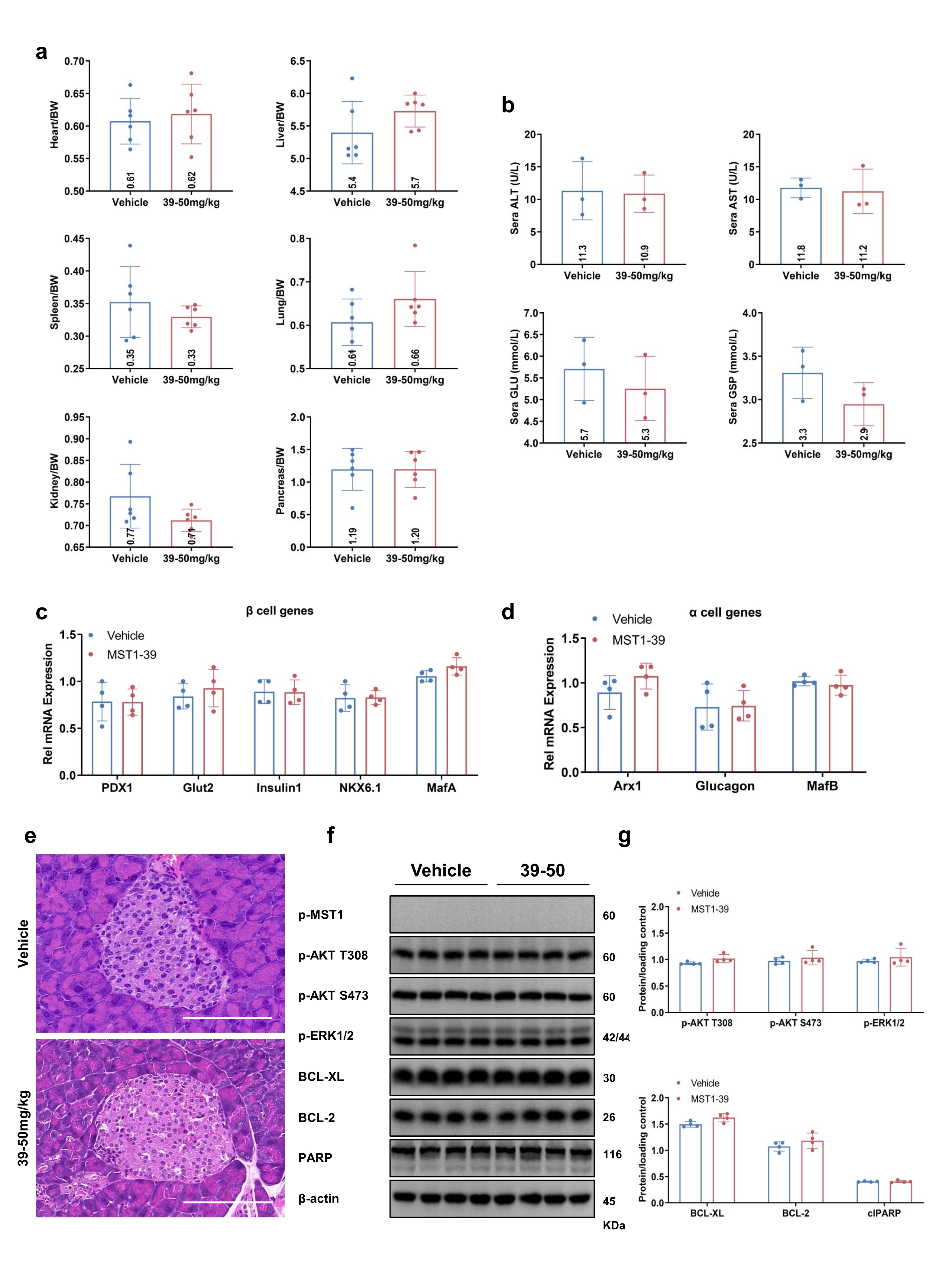


**Supplementary Figure S11** **a** Isolated tissues from C57BL6/J mice at day 80, the measurement of tissues weight normalized to body weight shown as change from vehicle group mice. **b** Serum AST, ALT, GLU, and GSP levels was measured by Chemray-240/800. **c-d** qPCR for PDX1, Glut2, Insulin1, NKX6.1, MafA, Arx1, Glucagon and MafB in C57BL6/J mice pancreatic tissues normalized to tubulin shown as change from vehicle group mice. **e** Representative images of HE stained pancreatic tissue sections showing pancreatic islets in all groups of experimental mice (scale bar = 50 μm). **f-g** IHMT-MST1-39 effect was analyzed by Immunoblot of pancreatic tissues. Immunoblot and densitometry analysis of p-MST1, p-AKT(T308), p-AKT(S473), p-ERK1/2, BCL-XL, BCL-2 and PARP. β-actin was used as loading control. C57 mice vehicle group (n = 4), C57 mice IHMT-MST1-39 group (n = 4). Data were expressed ± SEM. *p < 0.05, compound treated group compared with vehicle group mice; **p < 0.05, compound treated group compared with vehicle group mice; ***p < 0.001, compound treated group compared with vehicle group mice; P values determined by Student’s t test.

## Figure S12


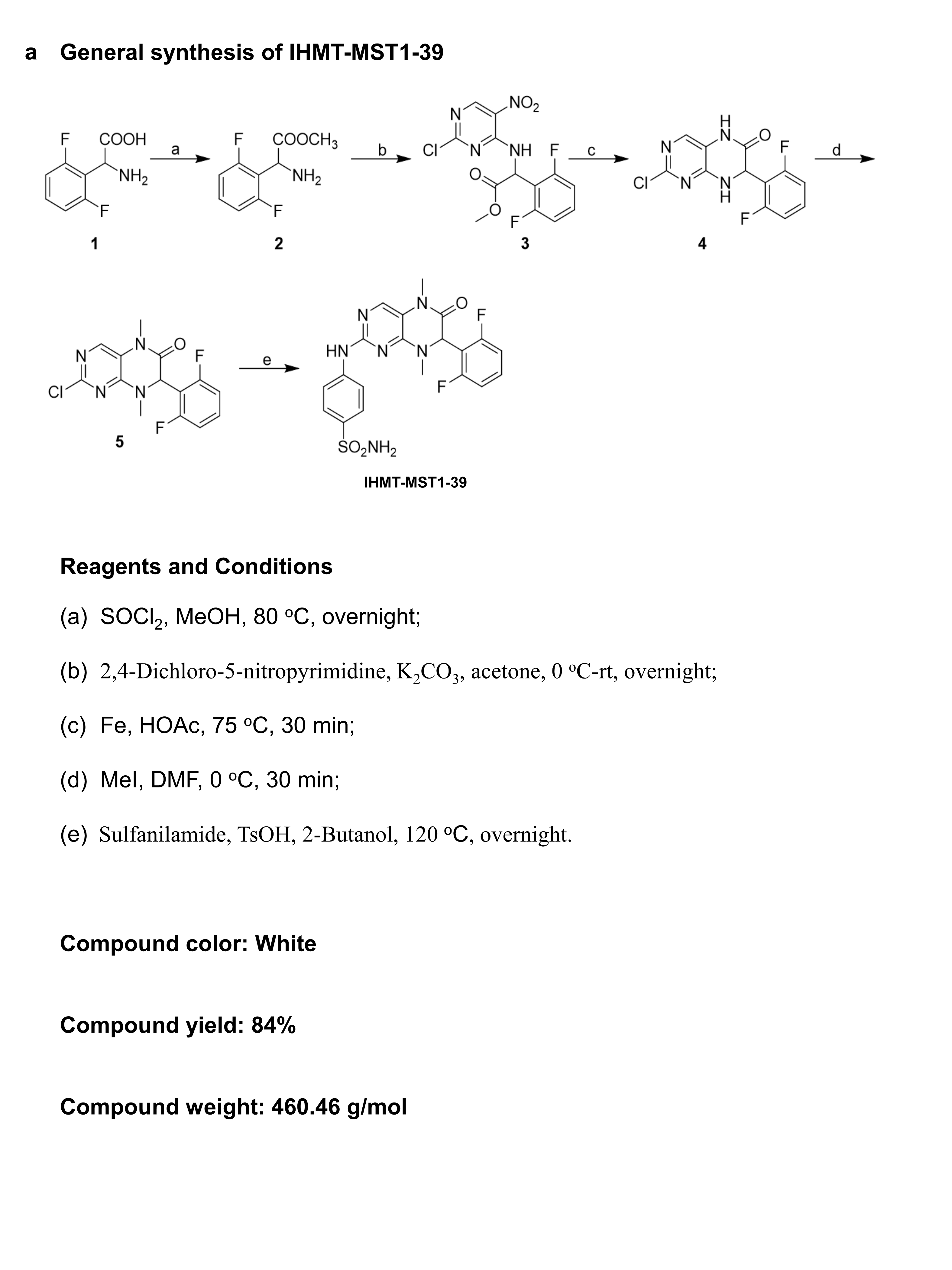


**Supplementary Figure S12** General synthesis of IHMT-MST1-39

## Figure S13


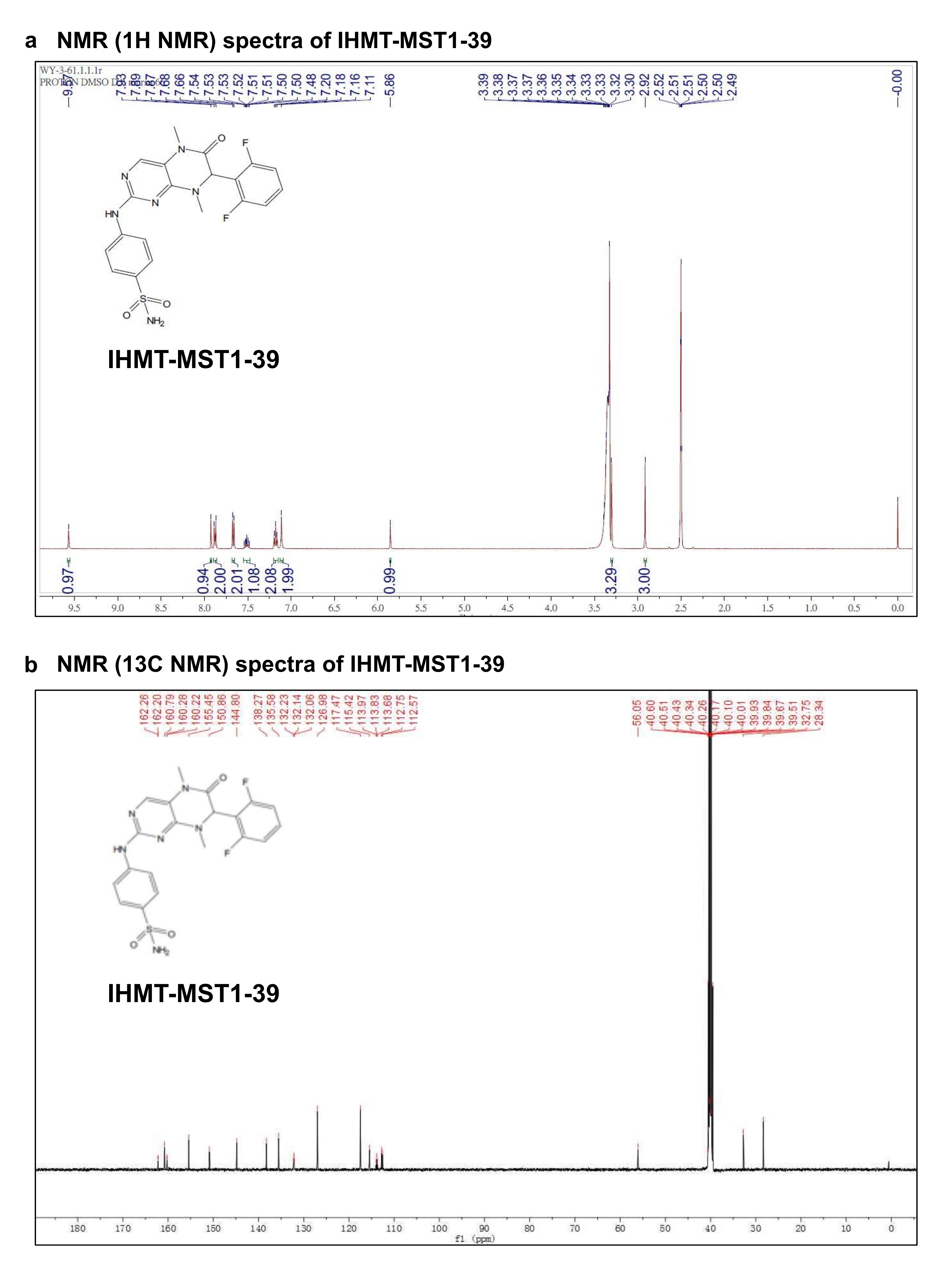


## Figure S14


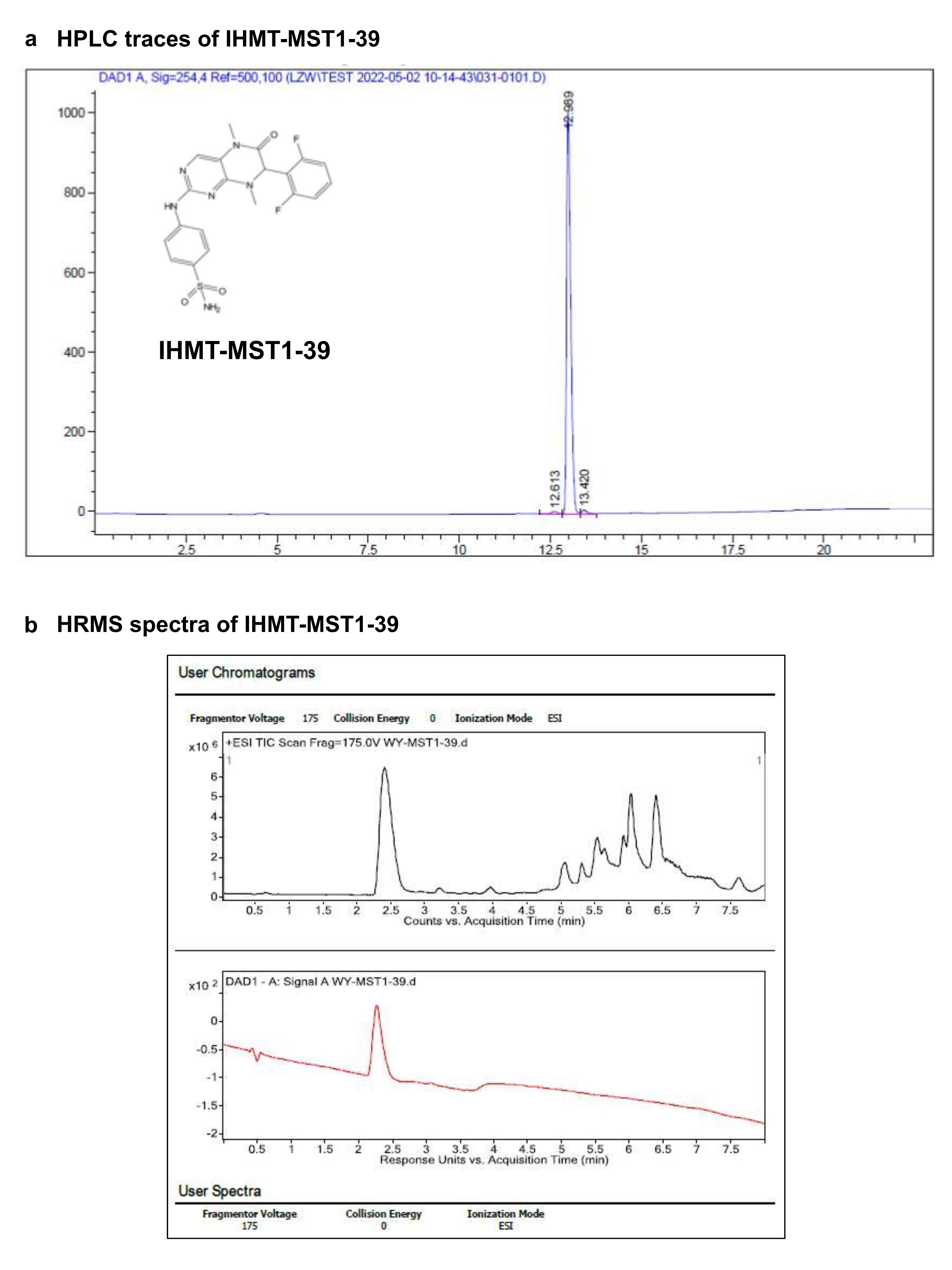


**Supplementary Table S1:**


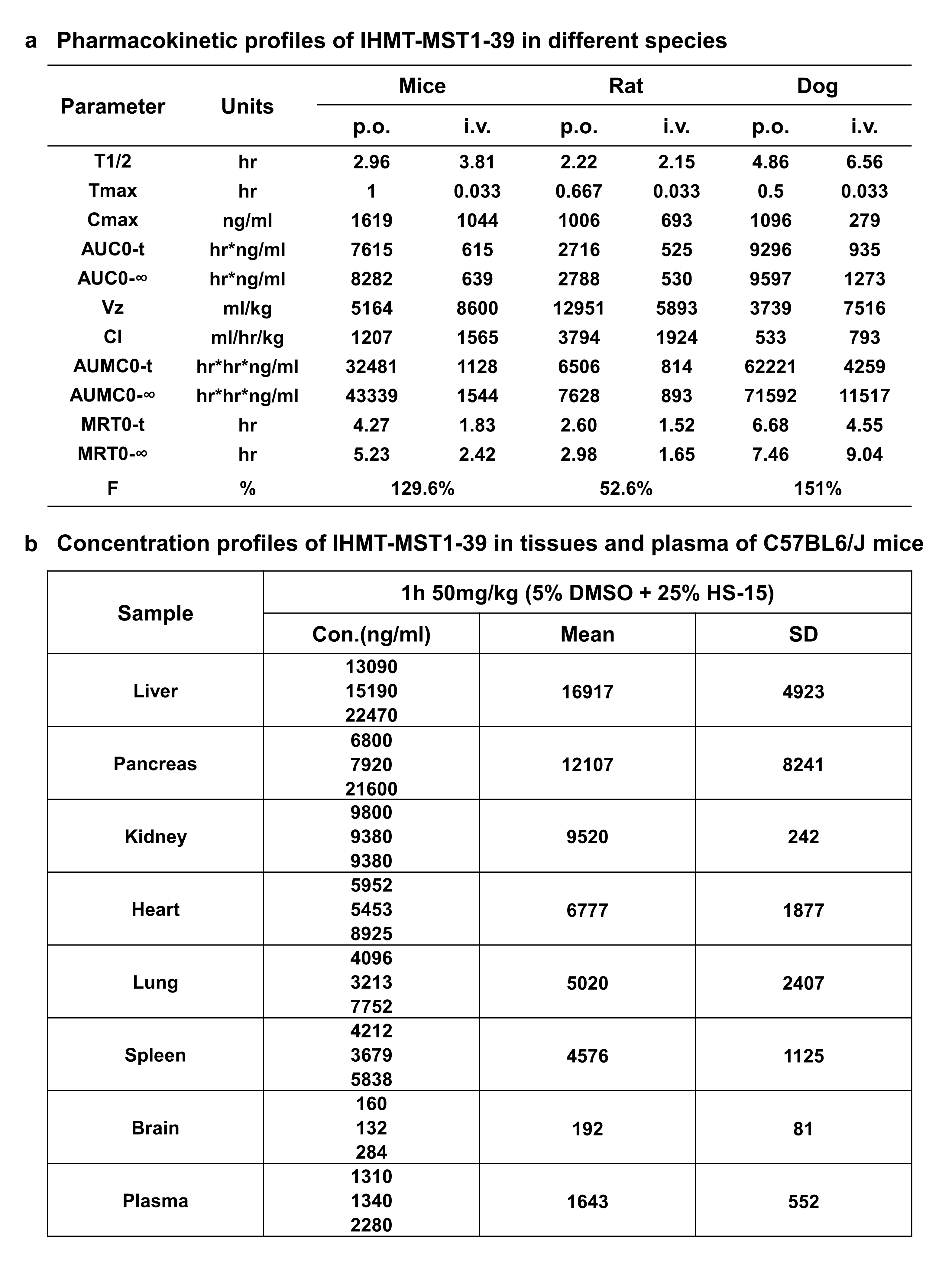


1 Wu, Y. *et al.* Discovery of 2-(4-Chloro-3-(trifluoromethyl)phenyl)-N-(4-((6,7-dimethoxyquinolin-4-yl)oxy)phen yl)acetamide (CHMFL-KIT-64) as a Novel Orally Available Potent Inhibitor against Broad-Spectrum Mutants of c-KIT Kinase for Gastrointestinal Stromal Tumors. *J Med Chem* **62**, 6083-6101 (2019).

2 Franzosa, E. A. *et al.* Gut microbiome structure and metabolic activity in inflammatory bowel disease. *Nat Microbiol* **4**, 293-305 (2019).

3 *Advanced Heatmap Plots was performed using the OmicStudio tools*, 2021.
